# PyHySCO: GPU-enabled susceptibility artifact distortion correction in seconds

**DOI:** 10.3389/fnins.2024.1406821

**Published:** 2024-05-27

**Authors:** Abigail Julian, Lars Ruthotto

**Affiliations:** ^1^Department of Computer Science, Emory University, Atlanta, GA, United States; ^2^Department of Mathematics, Emory University, Atlanta, GA, United States

**Keywords:** echo planar imaging, reversed gradient polarity, GPU acceleration, software, parallelization

## Abstract

Over the past decade, reversed gradient polarity (RGP) methods have become a popular approach for correcting susceptibility artifacts in echo-planar imaging (EPI). Although several post-processing tools for RGP are available, their implementations do not fully leverage recent hardware, algorithmic, and computational advances, leading to correction times of several minutes per image volume. To enable 3D RGP correction in seconds, we introduce PyTorch Hyperelastic Susceptibility Correction (PyHySCO), a user-friendly EPI distortion correction tool implemented in PyTorch that enables multi-threading and efficient use of graphics processing units (GPUs). PyHySCO uses a time-tested physical distortion model and mathematical formulation and is, therefore, reliable without training. An algorithmic improvement in PyHySCO is its use of the one-dimensional distortion correction method by Chang and Fitzpatrick to initialize the non-linear optimization. PyHySCO is published under the GNU public license and can be used from the command line or its Python interface. Our extensive numerical validation using 3T and 7T data from the Human Connectome Project suggests that PyHySCO can achieve accuracy comparable to that of leading RGP tools at a fraction of the cost. We also validate the new initialization scheme, compare different optimization algorithms, and test the algorithm on different hardware and arithmetic precisions.

## 1 Introduction

**Figure 1:**
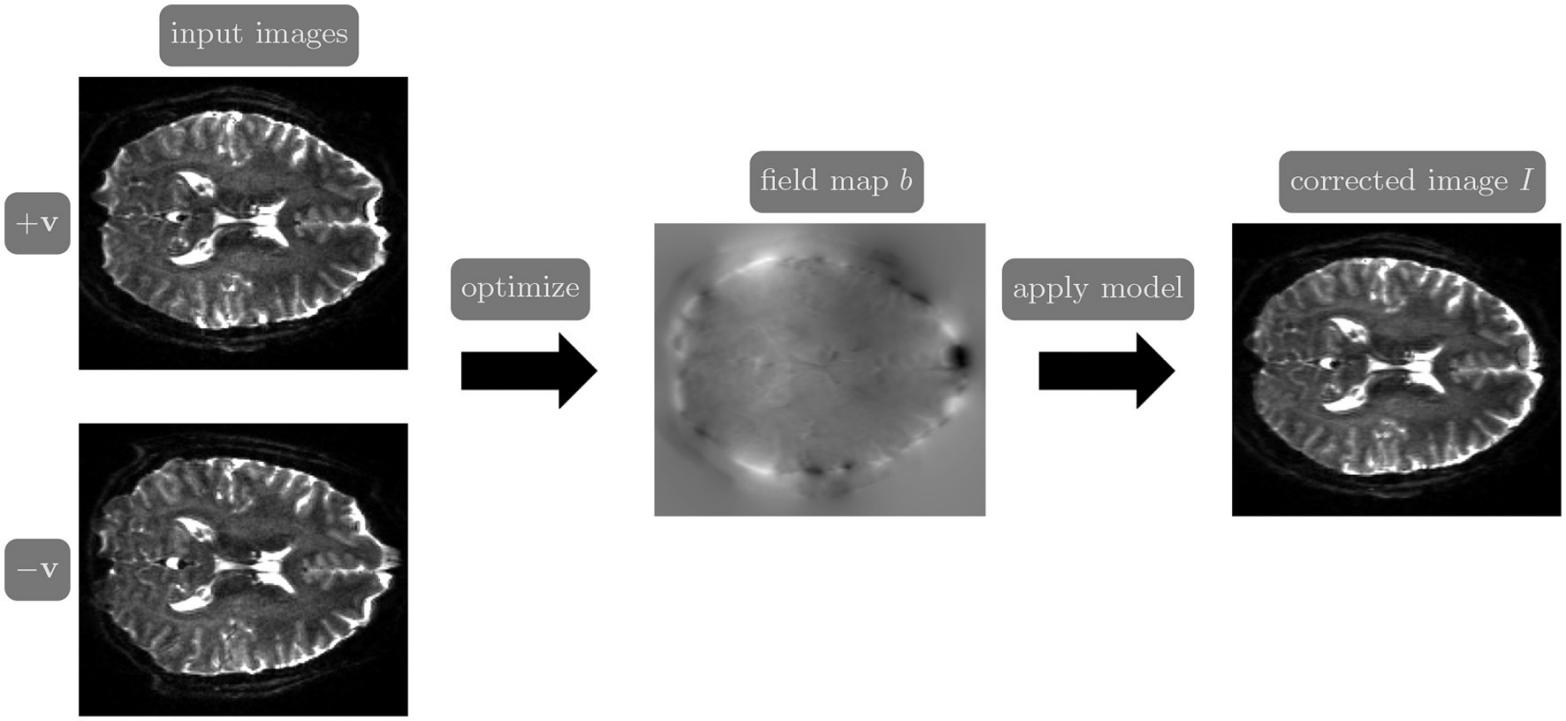
The reverse gradient polarity correction paradigm. Two images are acquired with opposite phase encoding directions, +**v** and −**v**. These two images are used to estimate the field map *b*, and the distortion correction model (Chang and Fitzpatrick, 1992) is applied to obtain a corrected image *I*.

Reversed gradient polarity (RGP) methods are commonly used to correct susceptibility artifacts in spin-echo echo-planar imaging (EPI; Stehling et al., 1991). RGP methods acquire a pair of images with opposite phase encoding directions, which leads to a minimal increase in scan time due to the speed of EPI. In a post-processing step, RGP approaches use the fact that the distortion in both images has an equal magnitude but acts in opposite directions to estimate the field map (see Figure 1; Chang and Fitzpatrick, 1992; Bowtell et al., 1994). The field map is then used to estimate a distortion-free image, either as a post-processing step using reconstructed images (Chang and Fitzpatrick, 1992) or simultaneously with image reconstruction by including the field map in the signal inverted during reconstruction (Zahneisen et al., 2017).

Compared to other correction approaches, such as field map acquisition, point-spread function map acquisition, and anatomical registration, RGP methods generally achieve comparable or superior accuracy while being more robust to noise and motion, see, e.g., Wu et al. (2008), Esteban et al. (2014), Graham et al. (2017), and Tax et al. (2022). These advantages make RGP correction a popular choice. For example, the widely-used MRI database from the Human Connectome Project (HCP; Van Essen et al., 2012) used the RGP correction tool TOPUP (Andersson et al., 2003) in the preprocessing of released diffusion MRI from EPI scans.

The original RGP distortion correction approaches in Chang and Fitzpatrick (1992) and Bowtell et al. (1994)'s studies are one-dimensional, treating each image column separately in the phase encoding direction. This leads to a non-smooth field map estimate and corrections. TOPUP addresses this non-smoothness with a 3D spline-based approach and the introduction of regularization (Andersson et al., 2003). TOPUP has limited support for hyperthreading and is often a time-consuming step of MRI processing pipelines (Cai et al., 2021). In our experiments, running TOPUP on a standard CPU took over 60 minutes on average per HCP subject.

Although less widely used than TOPUP, other iterative methods have proposed implementations of RGP correction employing various optimization schemes, discretizations, and regularization terms to speed up the correction. EPIC (Holland et al., 2010) introduces correction using a non-linear image registration framework. The tool was developed specifically for anterior-posterior distortions and can be less effective for left-right distortions (Gu and Eklund, 2019). DR-BUDDI (Irfanoglu et al., 2015) and TISAC (Duong et al., 2020a) methods regularize the optimization using either a T2-weighted or T1-weighted image, respectively. While including undistorted anatomical information can improve the quality of distortion correction (Gu and Eklund, 2019), it complicates the choice of an effective distance measure and, depending on the protocol, may require additional scan time. Hyperelastic Susceptibility Correction (HySCO) introduces hyper-elastic registration regularization and a novel separable discretization (Ruthotto et al., 2012, 2013; Macdonald and Ruthotto, 2017). HySCO can accurately correct real and simulated data varying in phase encoding direction, anatomy, and field of view (Gu and Eklund, 2019; Snoussi et al., 2021; Tax et al., 2022). In our experiments, on average, HySCO runs on the CPU for 1–2 min per HCP subject. While HySCO is a statistical parametric mapping (SPM; Penny et al., 2007) plugin and has been integrated into several SPM-based DTI processing pipelines, see, e.g., Clark et al. (2021) and Dávid et al. (2024), its dependency on a MATLAB license may limit its wider application.

Recently, several deep learning approaches for susceptibility artifact correction have been proposed due to extended processing times of the above mentioned RGP tools, see, e.g., Duong et al. (2020b, 2021), Hu et al. (2020), Zahneisen et al. (2020), and Alkilani et al. (2023). A recurrent theme is to train a correction operator in an offline stage in a supervised way using training data, which enables fast evaluations in the online step. For example, training S-Net on 150 volumes took over 5 days, while correcting an image pair on a CPU took an average of 2.8 s (0.96 s on a GPU; Duong et al., 2020b). However, the significant reduction of correction time comes at the cost of losing the robustness and generalizability that the existing RGP approaches obtain from the physical distortion model. For example, while RGP approaches can handle images from different scanners, anatomies, resolutions, and other acquisition parameters, deep learning models perform poorly when applied outside the training distribution (Chen et al., 2022). Furthermore, deep learning models are highly sensitive to noise and adversarial attacks in other contexts (Antun et al., 2020).

The PyHySCO (PyTorch Hyperelastic Susceptibility Correction) toolbox aims to achieve the accuracy, robustness, and generalizability of state-of-the-art RGP approaches at computational costs similar to evaluating a pre-trained deep learning model. PyHySCO offers EPI distortion correction through a GPU-enabled and command-line-accessible Python tool powered by PyTorch (Paszke et al., 2019). The mathematical formulation is based on HySCO augmented by a separable discretization (Macdonald and Ruthotto, 2017), which increases parallelism. PyHySCO alleviates the need for multilevel optimization by using the one-dimensional correction of Chang and Fitzpatrick (1992), which we derive through optimal transport. We demonstrate the use of PyHySCO using its Python interface and command-line tool, which is compatible with existing MRI postprocessing pipelines.

The remainder of our study is organized as follows. In Section 2, we review the mathematical model and its discretization under the hood of PyHySCO and describe the parallelized Chang and Fitzpatrick (CF) initialization using optimal transport, fast solvers exploiting the separable structure, and GPU-enabled PyTorch implementation. In Section 3, we extensively validate PyHySCO on real and simulated EPI data. We show the speed and accuracy of the CF initialization scheme and the speed and accuracy of the complete correction pipeline across optimizers, GPUs, and two levels of numerical precision. In Section 4, we discuss the benefits and implications of using PyHySCO for EPI distortion correction. In Section 5, we provide a conclusion.

## 2 Methods

This section describes the algorithmic and coding structure of PyHySCO. Section 2.1 introduces notation and reviews the mathematical formulation of the RGP correction problem. Section 2.2 describes the one-dimensional correction of Chang and Fitzpatrick (1992), which we use for initialization, and relates it to optimal transport. Section 2.3 describes the optimization algorithms available in PyHySCO. Section 2.4 explains the structure of the code and some key implementation details. Section 2.5 demonstrates the basic usage of PyHySCO and how to integrate it into existing processing pipelines.

### 2.1 Mathematical Formulation

The field map estimation and distortion correction are based on the physical forward model defined in Chang and Fitzpatrick (1992). Let **v** ∈ ℝ^3^ be the phase encoding direction for the distorted observation *I* : Ω → ℝ, and let Ω ⊂ ℝ^3^ be the image domain of interest. The mass-preserving transformation operator that, given the field map *b* : Ω → ℝ, corrects the distortions of an image *I* acquired with phase-encoding direction **v** reads


(1) $$\begin{array}{l} T\left[I,b,\text{v}\right]\left(\text{x}\right)=I\left(\text{x}+b\left(\text{x}\right)\text{v}\right)\cdot \left(1+\partial_{\text{v}}b\right)\left(\text{x}\right)\;\forall \text{x}\in \Omega . \end{array}$$

Here, ∂_**v**_*b* is the directional derivative of *b* in the direction of **v**. The first term of the operator corrects the geometric deformation in the direction of **v**, and the second is an intensity modulation term, which should always be positive.

Similar to Ruthotto et al. (2013), PyHySCO solves the inverse problem of estimating the field map *b* based on two observations, *I*_+**v**_ and *I*_−**v**_, acquired with phase-encoding directions ±**v**. To this end, we estimate the field map *b* by minimizing the distance of the corrected images.


(2) $$\begin{array}{l} D\left(b\right)=\frac{1}{2}\int_{\Omega }{\left(T\left[I_{+\text{v}},b,\text{v}\right]\left(\text{x}\right)-T\left[I_{-\text{v}},b,-\text{v}\right]\left(\text{x}\right)\right)}^{2}\;\text{d}\text{x}. \end{array}$$

The distance term is additionally regularized to enforce smoothness and the intensity modulation constraint. The smoothness regularization term


$$\begin{array}{l} S\left(b\right)=\frac{1}{2}\int_{\Omega }||\nabla b\left(\text{x}\right)|{|}^{2}\text{d}\text{x}, \end{array}$$

penalizes large values of the gradient of *b* to ensure smoothness in all directions.

The intensity modulation constraint of the physical model requires that −1 < ∂_**v**_*b*(**x**) < 1 for almost all **x** ∈ Ω. This is enforced by the barrier term as follows:


(3) $$\begin{array}{l} P\left(b\right)=\frac{1}{2}\int_{\Omega }\phi \left(\partial_{\text{v}}b\left(\text{x}\right)\right)\text{d}\text{x},\;\text{where }\phi \left(z\right)=\{\begin{array}{cc} \frac{z^{4}}{1-z^{2}}, & z\in \left(-1,1\right)\\ \infty , & \text{else.} \end{array} \end{array}$$

Altogether, this gives the optimization problem,


(4) $$\begin{array}{l} \underset{b}{\min}J\left(b\right)=D\left(b\right)+\alpha S\left(b\right)+\beta P\left(b\right), \end{array}$$

where the importance of the regularization terms is weighted with non-negative scalars α and β. Higher values of α promote a smoother field map, while lower values of α promote reduced distance between corrected images at the expense of smoothness in the field map. Any positive value for β ensures the intensity modulation constraint is satisfied, but lower values can lead to more ill-conditioned problems. For the purpose of this study, we fix α = 300 and β = 1e − 4.

PyHySCO follows the discretize-then-optimize paradigm commonly used in image registration, see, e.g., Modersitzki (2009). PyHySCO discretizes the variational problem (Equation 4) as in Macdonald and Ruthotto (2017) to obtain a finite-dimensional optimization problem almost entirely separable in the phase encoding direction. Specifically, coupling is only introduced in the smoothness regularization term when calculating the gradient in the frequency encoding and slice selection directions.

Our convention is to permute the dimensions of the input image such that the phase encoding direction is aligned with the third unit vector $e_{3}={\left[0,0,1\right]}^{T}$. The field map is discretized on an *e*_3_-staggered grid; that is, we discretize its values in the cell centers along the first two dimensions and on the nodes in the third dimension. The integrals in Equation (4) are approximated by a midpoint quadrature rule. The input images are modeled by a one-dimensional piecewise linear interpolation function in the phase encoding direction. The geometric transformation is estimated in the cell centers with an averaging operator, and the intensity modulation is estimated in the cell centers with a finite difference operator.

The discretized smoothness regularization term is computed for the discretized field map **b** via


(5) $$\begin{array}{l} S\left(\text{b}\right)=\frac{h_{1}\cdot h_{2}\cdot h_{3}}{2}{\text{b}}^{⊤}H\text{b}=\frac{h_{1}\cdot h_{2}\cdot h_{3}}{2}||\text{b}|{|}_{H}^{2}, \end{array}$$

where *h*_1_, *h*_2_, and *h*_3_ are the voxel sizes and *H* is a standard five-point discretization of the negative Laplacian and thus is a positive semi-definite operator. The discretized intensity modulation constraint term applies ϕ, as defined in Equation (3), element-wise to the result of a finite difference operator applied to the discretized field map. This results in the discretized optimization problem to be solved as follows:


(6) $$\begin{array}{l} \underset{\text{b}}{\min}J\left(\text{b}\right)=D\left(\text{b}\right)+\alpha S\left(\text{b}\right)+\beta P\left(\text{b}\right). \end{array}$$

This problem is challenging to solve because it is high-dimensional and non-convex, but we can exploit the structure and separability to efficiently solve the problem using parallelization. The implementation of this optimization problem in a parallelizable way, as described in Section 2.4, includes the choices of image interpolation, linear operators for averaging and finite difference, and regularization terms, *S* and *P*.

### 2.2 Parallelized one-dimensional initialization

Due to the non-convexity of the optimization problem (Equation 6), an effective initialization strategy for the field map is critical. To this end, PyHySCO initializes the correction with the result of the one-dimensional correction of Chang and Fitzpatrick (1992), which can be derived from optimal transport (OT) theory (Peyré and Cuturi, 2017). The key idea is to compute the ‘halfway' point of the oppositely distorted images in Wasserstein space (as opposed to Euclidean space, which would simply average the images). To render this problem feasible, we treat each image column separately, use the closed-form solutions of 1D OT problems, and then apply a smoothing filter. Implementing the Chang and Fitzpatrick (1992) correction using optimal transport provides a mathematical understanding of their algorithm and a highly accurate and parallelizable initialization.

We calculate these transformations as optimal transport maps (Peyré and Cuturi, 2017). More specifically, because the distortions only occur in the phase encoding direction, these transformations are a set of one-dimensional maps calculated in parallel across the distortion dimension. One-dimensional optimal transport has a closed-form solution considering the one-dimensional signal as a positive measure and constructing a cumulative distribution function (Peyré and Cuturi, 2017).

We describe the computation of the one-dimensional optimal transport maps in the distortion correction setting. In practice, the computation is parallelized in the distortion dimension to compute the entire initial field map simultaneously.

Let $i_{+\text{v}}\in {\mathbb{R}}^{m}$ be the image data from an entry in the phase encoding dimension of *I*_+**v**_, and let $i_{-\text{v}}\in {\mathbb{R}}^{m}$ be the image data from the corresponding entry in the phase encoding dimension of *I*_−**v**_. Consider *i*_half_ the sequence of image intensity values from the corresponding entry of the undistorted image *I*. We numerically ensure that *i*_+**v**_ and *i*_−**v**_ can be considered positive measures by applying a small shift to the image values, which does not change the relative distance between elements.

We initialize the field map using the optimal transport maps *T*_+_ from *i*_+**v**_ to *i*_half_ and *T*_−_ from *i*_−**v**_ to *i*_half_. These maps can be directly computed using the closed-form one-dimensional optimal transport formula, which depends on a cumulative distribution function and its pseudoinverse (Peyré and Cuturi, 2017).

We define the discretized cumulative distribution function *C*_*i*_:{0, …, *m*} → [0, 1] of a measure *i* as the cumulative sum as follows:


$$\begin{array}{l} \forall x\in \left\{0,\ldots ,m\right\}\;C_{i}\left(x\right)=\overset{x}{\underset{j=0}{\sum }}i\left(j\right), \end{array}$$

where *i*(*j*) returns the pixel intensity value at index *j* of *i*. The pseudoinverse $C_{i}^{-1}:\left[0,1\right]\to \left\{0,\ldots ,m\right\}$ is defined as follows:


$$\begin{array}{l} \forall r\in \left[0,1\right]\;C_{i}^{-1}\left(r\right)=\underset{x}{\min}\left\{x\in \left\{0,\ldots ,m\right\}\;|\;C_{i}\left(x\right)\geq r\right\}. \end{array}$$

In practice, $C_{i}^{-1}$ is computed using a linear spline interpolation.

**Figure 2:**
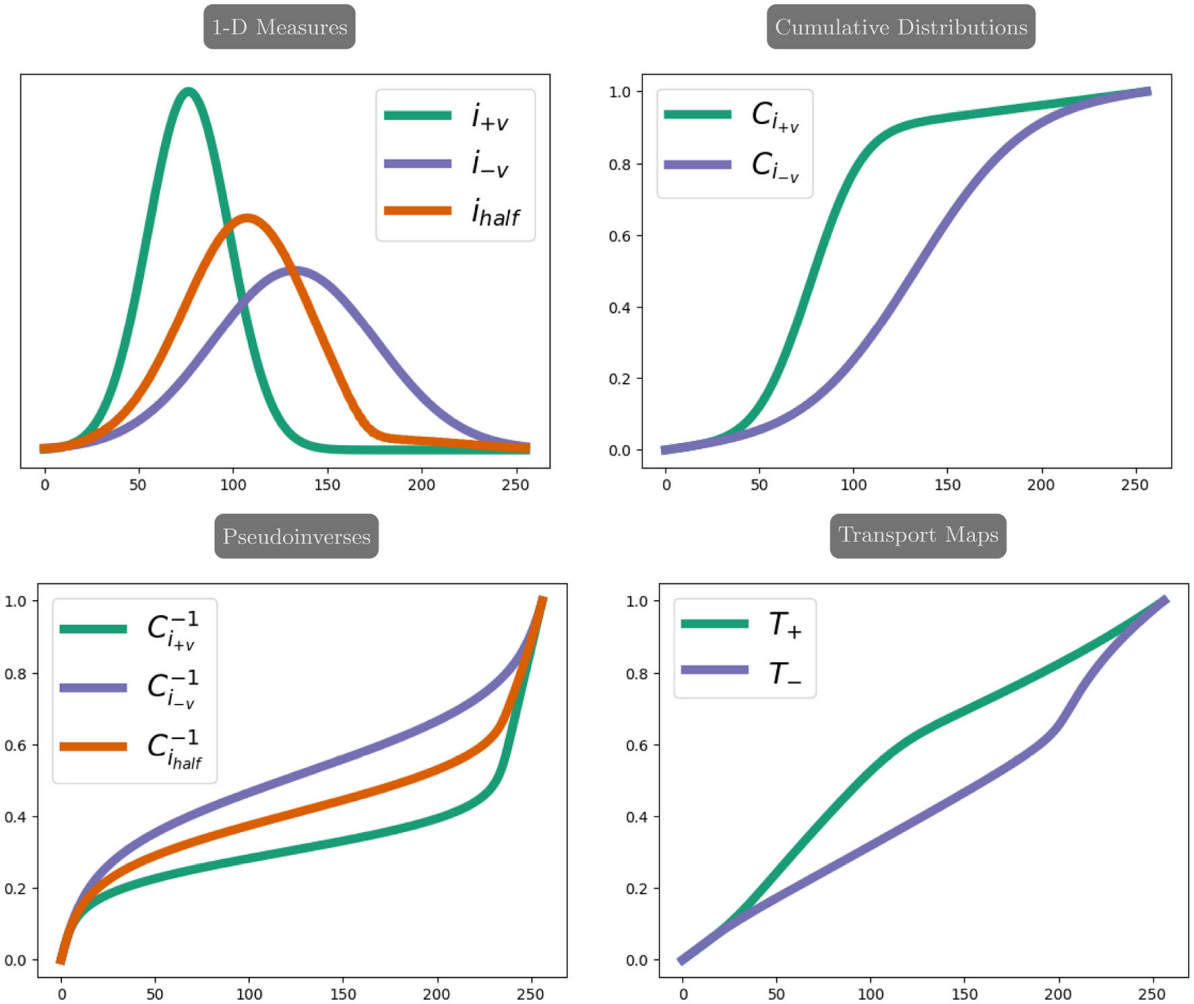
The example of one-dimensional optimal transport maps. The top left shows an example of one-dimensional measures. The green signal, *i*_+**v**_, corresponds to an intensity pileup in *I*_+**v**_, while the purple signal *i*_−**v**_ corresponds to an intensity dispersion in *I*_−**v**_. The red signal corresponds to the intensity of the true image. The top right shows the cumulative distributions for the measures *i*_+**v**_ and *i*_−**v**_. The bottom left shows the pseudoinverses for *i*_+**v**_ and *i*_−**v**_ along with the pseudoinverse $C_{i_{\text{half}}}^{-1}$ used in calculating the transport maps $T_{+}=C_{i_{\text{half}}}^{-1}\circ C_{i_{+\text{v}}}$ and $T_{-}=C_{i_{\text{half}}}^{-1}\circ C_{i_{-\text{v}}}$, which are shown in bottom right.

**Figure 3:**
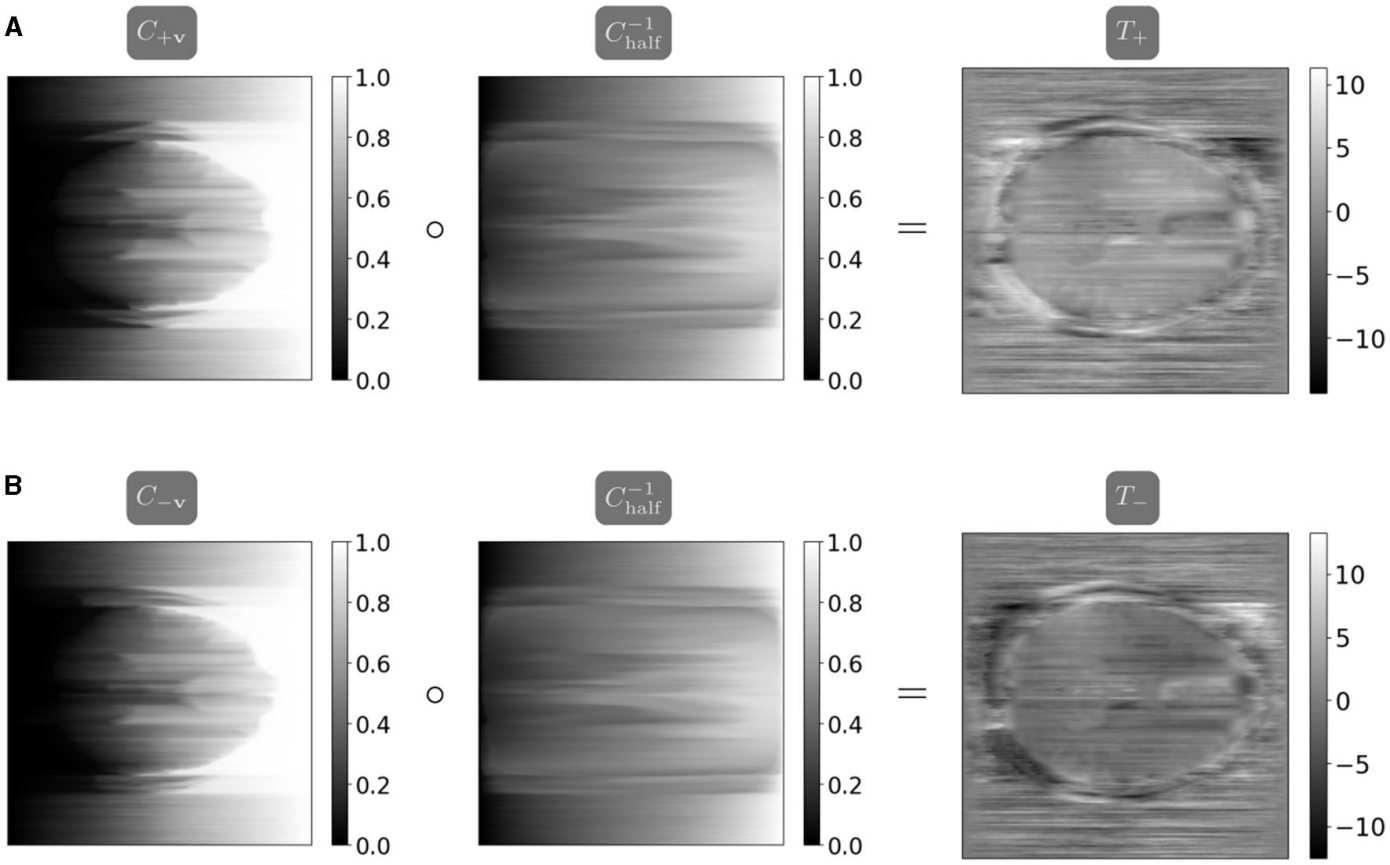
The maps *T*_+_ and *T*_−_ are calculated using the closed-form one-dimensional optimal transport solution, parallelized in the distortion dimension (Peyré and Cuturi, 2017). Note the inverted coloring between *T*_+_ and *T*_−_ as the map *T*_−_ corrects a distortion in the opposite direction as *T*_+_. **(A)** The map *T*_+_ mapping from *I*_+**v**_ halfway to *I*_−**v**_ is calculated as the composition of the cumulative distribution function *C*_+**v**_ from *I*_+**v**_ and the interpolated pseudoinverse $C_{\text{half}}^{-1}$. **(B)** The map *T*_−_ mapping from *I*_−**v**_ halfway to *I*_+**v**_ is calculated as the composition of the cumulative distribution function *C*_−**v**_ from *I*_−**v**_ and the interpolated pseudoinverse $C_{\text{half}}^{-1}$.

Returning to the measures arising from the input images, the closed-form solution for one-dimensional optimal transport gives the optimal transport map from *i*_+**v**_ to *i*_half_ as follows:


$$\begin{array}{l} T_{+}=C_{i_{\text{half}}}^{-1}\circ C_{i_{+\text{v}}}, \end{array}$$

and the optimal transport map from *i*_−**v**_ to *i*_half_ as follows:


$$\begin{array}{l} T_{-}=C_{i_{\text{half}}}^{-1}\circ C_{i_{-\text{v}}}, \end{array}$$

where $C_{i_{\text{half}}}^{-1}$ is calculated as $\left(C_{i_{+\text{v}}}^{-1}+C_{i_{-\text{v}}}^{-1}\right)/2$. Figure 2 visualizes the computation of the one-dimensional transport maps, and the parallelized computation and resulting field maps are visualized in Figure 3. We thus compute the initial guess for the field map as the average of the maps *T*_+_ and −*T*_−_, computed in parallel. We apply a smoothing filter to the initial field map before optimization to introduce smoothness in the frequency encoding and slice selection dimensions.

### 2.3 Optimization Algorithms

Since the optimal choice of optimization algorithms for approximately solving Equation (6) may depend on various factors, including image sizes, computational hardware, and severity of distortions, PyHySCO offers three options. Section 2.3.1 describes a Gauss-Newton scheme with a Jacobi-preconditioned conjugate gradient (GN-PCG) method as an inner solver, which is similar to Ruthotto et al. (2013) and is the default option. An option that exploits the parallelism of the discretization more effectively is the Alternating Direction Method of Multipliers (ADMM) in Section 2.3.2, which is based on Macdonald and Ruthotto (2017). For comparison, we also provide an interface to an LBFGS optimizer, see Section 2.3.3.

#### 2.3.1 GN-PCG: Gauss-Newton with Jacobi-Preconditioned Conjugate Gradient solver

PyHySCO's default solver is a PyTorch implementation of the GN-PCG scheme used in Ruthotto et al. (2013). Following the general idea of Gauss-Newton, we linearize the (non-linear) distortion correction operator (Equation 1) about the *k*-th iterate **b**_*k*_, obtain a quadratic model for the objective function by using a second-order Taylor approximation, and update the field map estimate with its approximate solution obtained with a few iterations of the PCG method.

More precisely, let ∇*J* be the gradient and *H*_*J*_ be a positive definite approximation of the Hessian of the optimization problem (Equation 6) about **b**_*k*_. Gauss-Newton iteratively updates the current field map estimate via


$$\begin{array}{l} {\text{b}}_{k+1}={\text{b}}_{k}+\gamma_{k}{\text{q}}_{k}, \end{array}$$

where the step size γ_*k*_ is determined using a line search method such as Armijo (Nocedal and Wright, 1999, Ch. 3 p. 33–36) and the search direction **q**_*k*_ approximately satisfies the following equation:


(7) $$\begin{array}{l} H_{J}\left({\text{b}}_{k}\right){\text{q}}_{k}=-\nabla J\left({\text{b}}_{k}\right). \end{array}$$

To obtain **q**_*k*_, we apply up to 10 iterations of the preconditioned conjugate gradient (PCG) method and stop early if the relative residual is less than 0.1, see the original work (Hestenes and Stiefel, 1952) or the textbook (Saad, 2003) for more details on PCG. The performance of PCG crucially depends on the clustering of the eigenvalues, which a suitable preconditioner can often improve. As a computationally inexpensive and often effective option, we implement a Jacobi preconditioner, which approximates the inverse of *H*_*J*_ by the inverse of its diagonal entries. Rather than constructing the matrix *H*_*J*_, which is computationally expensive, we provide efficient algorithms to compute matrix-vector products and extract its diagonal. While the diagonal preconditioner works well in our examples, we note that a more accurate (yet also more expensive) block-diagonal preconditioner has been proposed in Macdonald and Ruthotto (2017).

#### 2.3.2 Alternating Direction Method of Multipliers (ADMM)

We additionally modify the ADMM (Boyd et al., 2011) algorithm in Macdonald and Ruthotto (2017) and implement it in PyHySCO. To leverage separability of the objective function, the idea is to split the optimization problem into two subproblems. In contrast to Macdonald and Ruthotto (2017), which uses a hard constraint to ensure positivity of the intensity modulation and employs Sequential Quadratic Programming, we implement this as a soft constraint with the barrier term (Equation 3).

As in Macdonald and Ruthotto (2017), we split the objective in Equation (6) into


(8) $$\begin{array}{l} F\left(\text{b}\right)=D\left(\text{b}\right)+\alpha S_{3}\left(\text{b}\right)+\beta P\left(\text{b}\right),\;\text{and }G\left(\text{z}\right)=\alpha S_{1}\left(\text{z}\right)+\alpha S_{2}\left(\text{z}\right), \end{array}$$

where *S*_3_ is the part of the smoothness regularization term *S* corresponding to the phase encoding direction, and *S*_1_ and *S*_2_ are the remaining terms corresponding to the other directions. This gives rise to the following optimization problem, equivalent to Equation (6):


$$\begin{array}{l} \underset{\text{b},\text{z}}{\min}F\left(\text{b}\right)+G\left(\text{z}\right)\;\text{s.t. }\text{b}=\text{z}. \end{array}$$

With the corresponding augmented Lagrangian


$$\begin{array}{l} L\left(\text{b},\text{z},\text{y}\right)=F\left(\text{b}\right)+G\left(\text{z}\right)+{\text{y}}^{T}\left(\text{b}-\text{z}\right)+\frac{\rho h^{3}}{2}||\text{b}-\text{z}|{|}^{2}, \end{array}$$

where **y** is the Lagrange multiplier for the equality constraint **b** = **z** and ρ is a scalar augmentation parameter, and using scaled Lagrange multiplier $\text{u}=\frac{\text{y}}{\rho h^{3}}$, each iteration has the updates as follows:


(9) $$\begin{array}{l} {\text{b}}_{k+1}=\arg\underset{\text{b}}{\min}F\left(\text{b}\right)+\frac{\rho h^{3}}{2}||\text{b}-{\text{z}}_{k}+{\text{u}}_{k}|{|}^{2} \end{array}$$

(10) $$\begin{array}{l} {\text{z}}_{k+1}=\arg\underset{\text{z}}{\min}G\left(\text{z}\right)+\frac{\rho h^{3}}{2}||{\text{b}}_{k+1}-\text{z}+{\text{u}}_{k}|{|}^{2} \end{array}$$

(11) $$\begin{array}{l} {\text{u}}_{k+1}={\text{u}}_{k}+{\text{b}}_{k+1}-{\text{z}}_{k+1}. \end{array}$$

The **b** update computed in Equation (9) involves a separable optimization problem that can be solved independently for each image column along the phase-encoding direction. In PyHySCO, we use a modified version of the GN-PCG scheme described above. The only change is the computation of the search direction (Equation 7), which can now be parallelized across the different image columns. To exploit this structure, we implement a PCG method that solves the system for each image column in parallel. In addition to more parallelism, we observe an increase in efficiency since the scheme uses different step sizes and stopping criteria for each image column.

The **z** update is computed by solving the quadratic problem (Equation 10) directly. This update is enabled by the structure of the associated linear system, which is block-diagonal, and each block is given by a 2D negative Laplacian (from the regularizers) shifted by an identity (from the proximal term). Assuming periodic boundary conditions on the images, the blocks in the approximation itself have an exploitable structure [called Block Circulant—Circulant Block in Hansen et al. (2006)] and, therefore, can be inverted efficiently with the Fast Fourier Transform (FFT).

The scaled Lagrange multiplier **u** is updated at each iteration as in Equation (11). The augmentation parameter ρ is updated adaptively as described in Boyd et al. (2011) to keep the relative primal and dual residuals close.

#### 2.3.3 LBFGS

As a comparison, we provide an implementation of LBFGS (Liu and Nocedal, 1989), although optimization with LBFGS does not exploit any of the structure or separability of the optimization problem. LBFGS is a quasi-Newton method that uses an estimate of the objective function's Hessian based on a limited number of previous iterations in solving for the search direction (Liu and Nocedal, 1989). In our implementation, we provide an explicitly calculated derivative to an LBFGS solver.^1^ While computing the objective function, we precompute parts of the derivative which allows for faster optimization than relying on automatic differentiation.

### 2.4 Coding structure of PyHySCO

**Figure 4:**
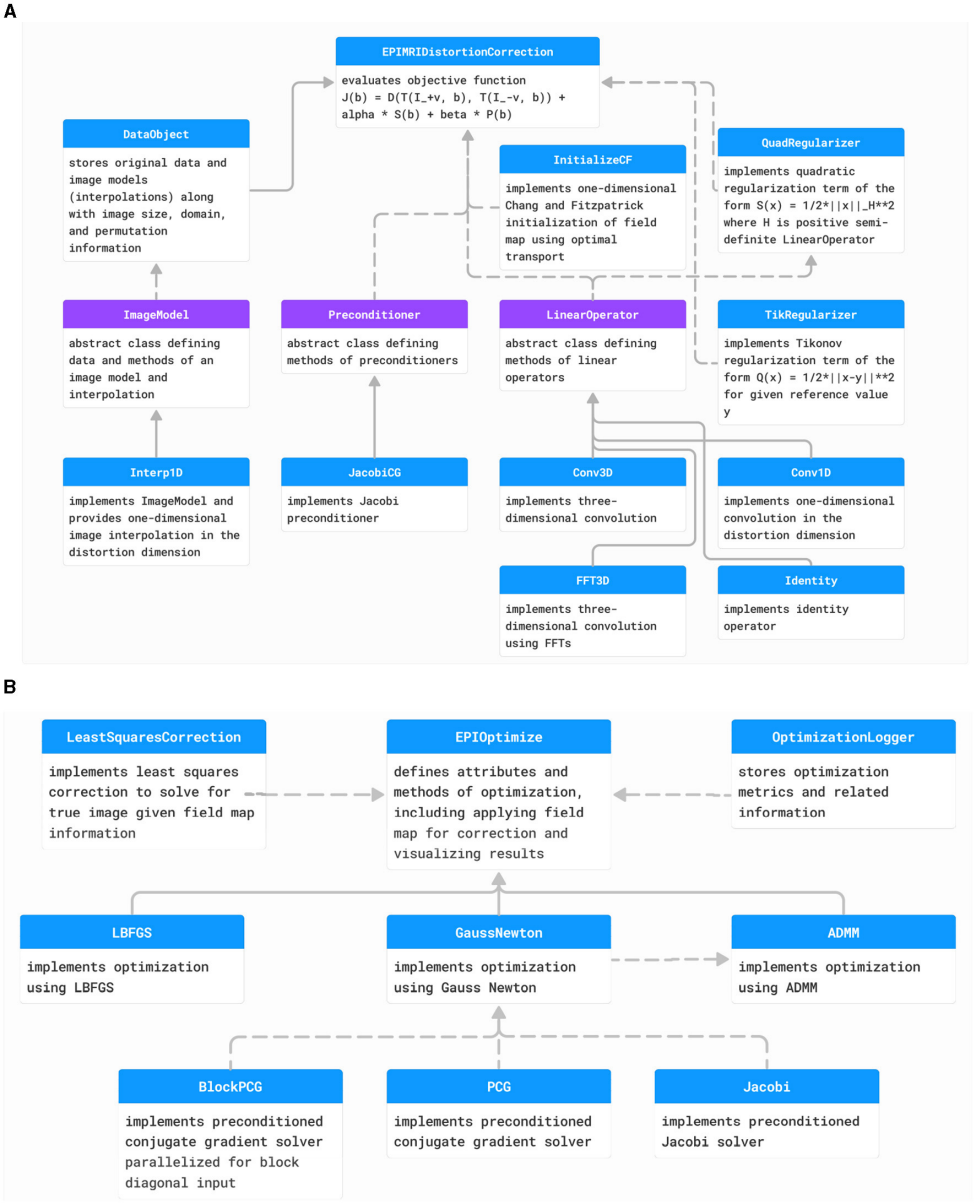
**(A)** The class structure of the PyHySCO loss function. The main class representing the loss function is EPIMRIDistortionCorrection. Purple classes are abstract, and blue classes are concrete. Solid arrows indicate inheritance. Dashed arrows indicate dependencies and class objects that are attributes. **(B)** The class structure of PyHySCO optimization. The main class defining optimization is EPIOptimize. Solid arrows indicate inheritance. Dashed arrows indicate dependencies and class objects that are attributes. UML diagram of PyHySCO showing the classes and relationships for the **(A)** loss function and **(B)** optimization. A EPIMRIDistortionCorrection object defining the loss function is an attribute of every EPIOptimize object defining the optimization scheme.

We implemented PyHySCO in PyTorch (Paszke et al., 2019) following the overall code structure visualized in the diagrams in Figures 4A, B for the objective function and optimization, respectively. The main classes of PyHySCO are the loss function, which is implemented in EPIMRIDistortionCorrection, and the optimization, which is defined in EPIOptimize. The other classes and methods, described in detail in the following, implement the components of the loss function evaluation and optimization schemes.

#### 2.4.1 Data storage and the image model

The input pair of images with opposite phase encoding directions are loaded and permuted such that the distortion dimension is the last, as this is where PyTorch expects the batch dimension for parallelizing operations. Information on the input images is stored in an object of type DataObject. This class stores information on the image size, domain, voxel size, how to permute the data back to the input order, and the ImageModel for each input image. The ImageModel abstract class defines the structure and required methods for an image model, including storing the original data and providing a method eval that returns the data interpolated on the given points. We provide the default implementation Interp1D, a piecewise linear one-dimensional interpolation parallelized in the last dimension. The DataObject for a given input pair is then stored in the EPIMRIDistortionCorrection object.

#### 2.4.2 The correction model

The mass-preserving correction model (Equation 1) is implemented in the method mp_transform, a class method of EPIMRIDistortionCorrection. The method takes as input an ImageModel and a field map. The geometric deformation is computed by using an averaging LinearOperator to compute the field map values in the cell centers and adding this to a cell-centered grid to obtain the deformed grid defined by this field map. Using the ImageModel, the image is interpolated on this deformed grid. The intensity modulation term is computed using a finite difference LinearOperator. The two terms are multiplied together element-wise before returning the corrected image. The default implementation of the LinearOperator objects for averaging and finite difference are given as one-dimensional convolutions, parallelized in the last dimension.

#### 2.4.3 Regularization terms

The intensity regularization term is computed within the EPIMRIDistortionCorrection class in the method phi_EPI which computes the result of applying ϕ, as defined in Equation (3), element-wise to the result of applying the finite difference operator to the field map, as computed in the correction model. This function acts as a barrier term, ensuring that the derivative of the field map in the distortion dimension is in the range (–1, 1).

The smoothness regularization term is implemented in a QuadRegularizer object, which defines the evaluation of a quadratic regularization term of the form of Equation (5) using a positive semi-definite LinearOperator as *H*. By default, *H* is a discretized negative Laplacian applied via a three-dimensional convolution.

In the ADMM optimizer, the regularizer structure differs to account for the splitting in Equation (8). The objective function for the **b** update in Equation (9) is computed in EPIMRIDistortionCorrection where the computation of *S*_3_ is a one-dimensional Laplacian in the distortion dimension applied via a one-dimensional convolution. The proximal term is computed through a TikRegularizer object, a Tikhonov regularizer structure. The objective function for the **z** update in Equation (10) is a QuadRegularizer object, where the LinearOperator *H* is a two-dimensional Laplacian corresponding to *S*_2_ and *S*_3_. This operator is implemented in FFT3D, which defines an operator applying a convolution kernel diagonalized in Fourier space (Cooley et al., 1969). This implementation allows for easily inverting the kernel while solving for **z**.

#### 2.4.4 Hessian and preconditioning

For the Gauss-Newton and ADMM optimizers, an approximate Hessian and preconditioner are additionally computed. The parts of the Hessian are computed in EPIMRIDistortionCorrection during objective function evaluation, and the Hessian can be applied through a matrix-vector product. Similarly, a Preconditioner can be computed during objective function evaluation and is accessible through a returned function applying the preconditioner to its input. By default, we provide a Jacobi preconditioner in the class JacobiCG.

#### 2.4.5 Initialization

The EPIMRIDistortionCorrection class has a method initialize, returning an initial guess for the field map using some InitializationMethod. We provide an implementation of the proposed parallelized Chang and Fitzpatrick initialization in InitializeCF. The implementation computes the one-dimensional transport maps in parallel using a linear spline interpolation. In practice, the parallelized initialization gives a highly non-smooth initial field map, so the method optionally applies a blurring operator using a 3-by-3-by-3 Gaussian kernel with a standard deviation of 1.0 to promote a smoother optimized field map. Applying the blur to the field map is implemented using the fast FFT convolution operator FFT3D.

#### 2.4.6 Optimization

The minimization of the objective function defined in a EPIMRIDistortionCorrection object happens in a subclass of EPIOptimize, which takes the objective function object as input. During optimization, the OptimizationLogger class is used to track iteration history, saving it to a log file and optionally printing this information to standard output. PyHySCO includes implementations of the LBFGS, Gauss-Newton, and ADMM solvers described previously. Each of the classes LBFGS, GaussNewton, and ADMM provide a run_correction method that minimizes the objective function using the indicated optimization scheme. The LBFGS implementation uses the explicitly computed derivative from EPIMRIDistortionCorrection. For LBFGS, we use the norm of the gradient reaching a given tolerance as stopping criteria, or the change in loss function or field map between iterations falling below a given tolerance. The GaussNewton implementation uses a conjugate gradient solver implemented in the class PCG. Our Gauss-Newton implementation uses the same stopping criteria as LBFGS. The ADMM implementation solves the **b** update in Equation (9) using GaussNewton with a parallelized conjugate gradient solver in BlockPCG. The **z** update in Equation (10) is solved directly through the inverse method inv of the operator used to define the QuadRegularizer for this term, efficiently implemented using FFTs in FFT3D. As stopping criteria, the ADMM iterations will terminate if the change in all of **b**, **z**, and **u** from the previous iteration falls below a given tolerance.

#### 2.4.7 Image correction

The optimal field map, stored as Bc in the EPIOptimize object after run_correction is completed, can be used to produce a corrected image or pair of images. The apply_correction method of EPIOptimize implements both a Jacobian modulation correction and a least squares correction. The Jacobian modulation correction is based on the model of Chang and Fitzpatrick (1992) as implemented in the mp_transform method of EPIMRIDistortionCorrection. This correction method computes and saves two corrected images, one for each input image.

The field map can also be used in a least squares correction similar to the correction in Andersson et al. (2003), implemented in LeastSquaresCorrection. In this correction, the estimated field map determines a push-forward matrix that transforms the true image to the distorted image given as input. This gives rise to a least squares problem for the true image, given the input images and push forward matrix.

### 2.5 PyHySCO usage and workflow

**Figure 5:**
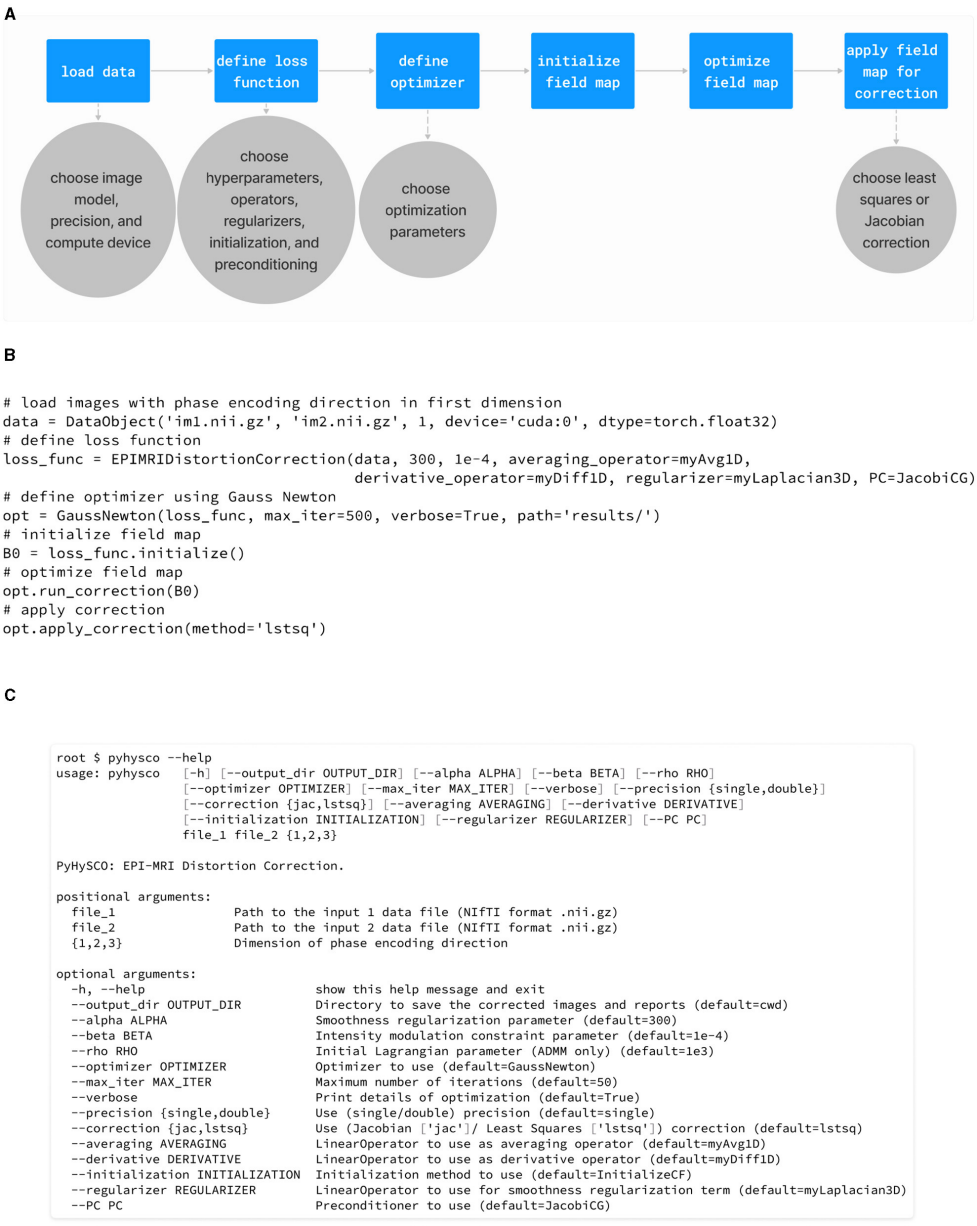
**(A)** The workflow of the PyHySCO toolbox from setup through optimization and distortion correction. **(B)** An example of using the PyHySCO toolbox from a Python script. **(C)** The help message for the PyHySCO command line interface. This interface allows the use of PyHySCO as a part of existing MRI post-processing pipelines. The usage and workflow of PyHySCO.

The workflow of PyHySCO is illustrated in Figure 5A alongside examples of using PyHySCO in a Python script (Figure 5B) and through the command line (Figure 5C). Running PyHySCO from a user-defined Python script allows for more control of the inputs and outputs from PyHySCO methods. The command line interface allows the user to pass configuration options directly from the command line, which enables our EPI distortion correction tool to be easily used as a part of the existing command line based MRI post-processing pipelines such as the FMRIB Software Library (FSL) toolbox (Smith et al., 2004). Executing PyHySCO requires the user to provide, at a minimum, the file paths for the input pair of images with opposite phase encoding directions and which dimension (1, 2, or 3) is aligned with the phase encoding direction. The modularity of PyHySCO additionally allows for configuring options such as the scalar hyperparameters in Equation (6); implementation of operators, regularizers, and interpolation; optimizer and associated optimization parameters; and the image correction method.

Regardless of execution through a script or the command line, PyHySCO stores the input images in a DataObject object, the loss function in an EPIMRIDistortionCorrection object, and the optimizer in an object of a subclass of EPIOptimize. The field map is initialized from the method initialize in EPIMRIDistortionCorrection, and the field map is optimized by calling the method run_correction in the optimizer object. Finally, the method apply_correction in EPIOptimize applies the field map to correct the input images and saves the result to one or more NIFTI file(s).

## 3 Results

We demonstrate PyHySCO's effectiveness through extensive experiments using real and simulated data from the Human Connectome Project (Van Essen et al., 2012) and validate the initialization scheme and the implementation of optimization algorithms. Section 3.1 describes the datasets and Section 3.2 introduces our evaluation metrics. Section 3.3 demonstrates the Chang and Fitzpatrick initialization scheme. The experiments in Section 3.4 compare the performance of the three optimization algorithms implemented in PyHySCO on CPU and GPU hardware. Section 3.5 compares the performance of PyHySCO in single and double-precision arithmetic on CPU and GPU hardware. Section 3.6 compares PyHySCO with existing tools, HySCO and TOPUP (Andersson et al., 2003; Ruthotto et al., 2013).

### 3.1 Validation datasets

The data used in the following experiments is from the Human Connectome Project (Van Essen et al., 2012). We validate our methods and tool on 3T and 7T diffusion-weighted imaging data from the HCP 1200 Subjects Release, with 20 subjects randomly chosen for each field strength. Table 1 provides details of the datasets.

**Table 1:** Details of data used in validation. LR/RL is left-to-right and right-to-left phase encoding, and AP/PA is anterior-to-posterior and posterior-to-anterior phase encoding.

**Dataset**	**No. of subjects**	**Image size**	**Resolution**	**PE directions**
3T	20	168 × 144 × 111	1.25 × 1.25 × 1.25 *mm*^3^	LR/ RL
7T	20	200 × 200 × 132	1.05 × 1.05 × 1.05 *mm*^3^	AP/ PA
Simulated	20	320 × 320 × 256	0.7 × 0.7 × 0.7 *mm*^3^	AP/ PA

Further details of acquisition parameters are in Van Essen et al. (2012).

We also evaluate our methods on simulated data. This data only contains susceptibility artifact distortions, thus it shows how our tool performs without the influence of other factors, e.g., patient movement between scans. To simulate the distortions, we use a pair of magnitude and phase images for a subject in HCP and generate the field map using FSL's FLIRT and PRELUDE tools (Smith et al., 2004). Considering the physical model of Chang and Fitzpatrick (1992), the field map *b* can be used to define the push-forward matrices that show how the intensity value at **x** is pushed forward to **x**+*b*(**x**) in the distortion direction +**v** as well as the opposite direction −**v**. By applying the push-forward matrices to a T2-weighted image for the subject, we generate a pair of distorted images. For the simulated data, we then have a reference value for the field map and an undistorted, true image.

### 3.2 Metrics

The quality of correction results is measured using the relative improvement of the distance between a pair of corrected images. Particularly, we calculate the sum-of-squares distance (SSD, Equation 2) of the corrected image pair relative to the SSD of the input pair. This metric is a useful surrogate for the correctness of the field map in the absence of a ground truth (Graham et al., 2017). Additionally, we take the value of the smoothness regularization term *S*(**b**) as a measure of how smooth the resulting field map is, with lower values being better.

We report the runtime in seconds of PyHySCO. The runtime is measured as the wall clock time using the Linux time command when calling the correction method from the command line. This time, therefore, includes the time taken to load and save the image data. In some cases, we also report the optimization time only, without loading and saving data, as measured by Python's time module.

### 3.3 Validity of Chang and Fitzpatrick initialization

**Figure 6:**
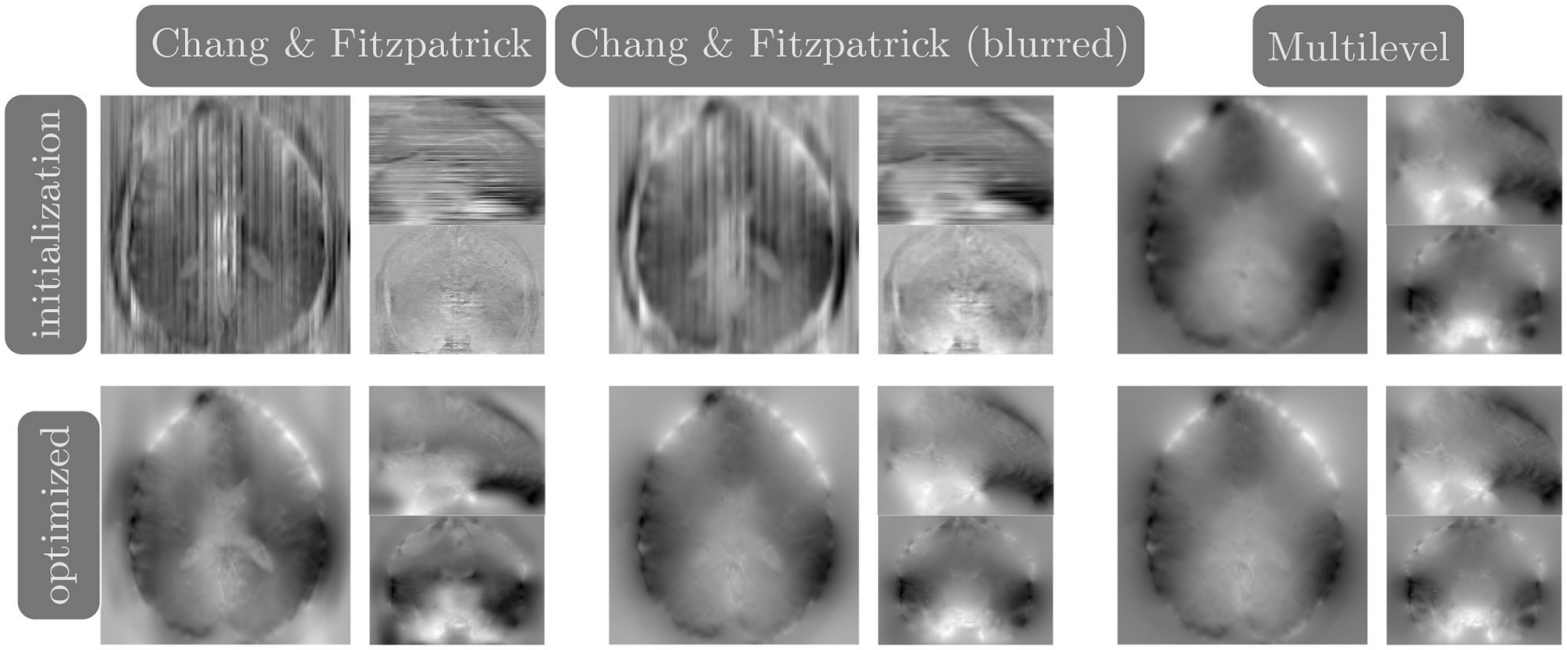
Example field maps (Subject ID 826353) at initialization **(top row)** and after optimization with Gauss-Newton **(bottom row)**. The first column uses the proposed Chang and Fitzpatrick initialization scheme. The middle column uses the same scheme with an additional Gaussian blur to promote smoothness. The right column uses the coarse-to-fine multilevel initialization scheme from HySCO with five levels, and the final field map is optimized at the original image resolution. The multilevel initialized field map is smooth by construction and further optimized to improve the relative image distance at the full resolution. The Chang and Fitzpatrick initialization accurately corrects the distortions but is not smooth in the non-distortion dimensions unless blurred with a Gaussian. After the fine-level optimization, all field maps are visually similar.

We compare the results of PyHySCO using the one-dimensional parallelized Chang and Fitzpatrick initialization to those of the multi-level initialization used in HySCO (Ruthotto et al., 2013) both at initialization and after optimization with Gauss-Newton. The multi-level optimization of HySCO solves the optimization problem on a coarse grid and uses the result as the initialization of optimization on a finer grid, continuing until the original image resolution is reached; this follows the guidelines of Modersitzki (2009, Chapter 9.4). In our experiments, we use five levels of initialization. The multi-level initialization gives a field map that is smooth by construction and improves the distance reduction as the grid becomes more fine. The field map from the PyHySCO Chang and Fitzpatrick initialization drastically lowers the relative error between the input images, a relative improvement of over 96% on real data and 94% on simulated data. However, the parallelized one-dimensional computations lead to a lack of smoothness in the resulting field map. The smoothness can be improved by applying a Gaussian blur to the field map from the Chang and Fitzpatrick initialization. This field map is smoother after initialization and gives a smoother field map after optimization. These results are comparable in relative error and smoothness to the field map optimized using the multilevel initialization of HySCO. Our one-dimensional parallelized initialization, even if adding additional Gaussian blur, is much faster to compute than the multilevel initial field map, given the ability to parallelize computations. PyHySCO initialization on a GPU with the additional blur takes less than 1 s on real data and ~3 s on simulated data. In comparison, the multi-level initialization on a CPU takes 30–40 s on real data and over 2 min on simulated data. The mean and standard deviation of relative improvement, smoothness value, loss function value, and runtime are reported in Table 2 across all datasets. The examples of these field maps before and after optimization are shown in Figure 6.

**Table 2:** Validation of the Chang and Fitzpatrick initialization.

		**Chang and Fitzpatrick**		**Chang and Fitzpatrick (blur)**		**Multilevel**	
		**Initial**	**After opt**	**Initial**	**After opt**	**Initial**	**After opt**
3T	Runtime (s)	5.78	11.43	6.31	15.36	41.69	55.34
		±1.26	±1.46	±0.60	±3.90	±1.71	±2.84
	Opt. Time (s)	0.27	4.34	0.28	6.78	42.43	48.65
		±0.01	±0.67	±0.02	±0.67	±4.04	±3.95
	Relative	96.44	83.90	79.71	82.75	67.04	81.96
	Improvement	±1.13	±3.43	±3.43	±3.49	±5.15	±3.51
	Loss Value	1.05*e*09	2.84*e*07	1.76*e*08	2.56*e*07	4.82*e*07	2.51*e*07
		±2.66*e*08	±7.49*e*06	±5.20*e*07	±7.66*e*06	±1.70*e*07	±7.54*e*06
	Smoothness	3.50*e*06	5.08*e*04	5.28*e*05	3.85*e*04	6.89*e*04	3.47*e*04
	Reg. Value	±8.81*e*05	±1.23*e*04	±1.54*e*05	±1.21*e*04	±2.98*e*04	±1.08*e*04
7T	Runtime (s)	7.61	13.55	8.32	19.72	58.73	77.79
		±1.99	±2.04	±2.79	±2.91	±6.24	±5.72
	Opt. Time (s)	0.61	5.09	0.63	10.16	30.38	40.50
		±0.02	±1.23	±0.02	±0.85	±2.63	±3.87
	Relative	96.53	86.01	75.09	85.76	69.12	85.42
	Improvement	±1.47	±5.15	±3.97	±5.10	±8.28	±5.08
	Loss Value	3.48*e*09	5.28*e*07	4.50*e*08	4.14*e*07	7.77*e*07	4.02*e*07
		±1.15*e*09	±2.01*e*07	±2.47*e*08	±1.95*e*07	±3.01*e*07	±1.82*e*07
	Smoothness	1.16*e*07	9.52*e*04	1.36*e*06	5.63*e*04	8.21*e*04	5.03*e*04
	Reg. Value	±3.83*e*06	±2.64*e*04	±7.74*e*05	±1.91*e*04	±4.07*e*04	±1.48*e*04
Simulated	Runtime (s)	10.62	80.29	16.59	106.47	173.20	47.98
		±0.57	±9.96	±0.64	±11.21	±27.06	±8.38
	Opt. Time (s)	3.51	64.45	3.61	89.17	125.35	157.95
		±0.03	±10.02	±0.15	±11.48	±24.88	±28.79
	Relative	94.64	76.82	75.34	76.27	55.01	73.63
	Improvement	±1.26	±5.09	±3.44	±5.18	±5.66	±5.39
	Loss Value	5.10*e*08	6.31*e*07	2.11*e*08	6.07*e*07	8.17*e*07	5.83*e*07
		±9.51*e*07	±1.46*e*07	±4.30*e*07	±1.39*e*07	±2.08*e*07	±1.33*e*07
	Smoothness	1.67*e*06	1.06*e*05	5.84*e*05	9.53*e*04	6.18*e*04	7.50*e*04
	Reg. Value	±3.14*e*05	±2.94*e*04	±1.24*e*05	±2.71*e*04	±1.70*e*04	±2.20*e*04

We compare the runtime, relative improvement, smoothness value, and loss function value at initialization and after optimization with Gauss-Newton for the proposed parallelized Chang and Fitzpatrick initialization, the proposed initialization with an additional Gaussian blur, and the multilevel initialization used in HySCO (Ruthotto et al., 2013). For each metric, we report the mean and standard deviation in the 3T, 7T, and simulated datasets. The multilevel initialization is timed on CPU in Matlab, and the Chang and Fitzpatrick initializations and all optimizations are timed on GPU in Python. The Chang and Fitzpatrick-based initializations provide a comparable quality while decreasing runtime compared to the multilevel initialization, and the Chang and Fitzpatrick with Gaussian blur promotes a smoother field map.

### 3.4 A comparison of PyHySCO Optimizers on GPU and CPU

**Figure 7:**
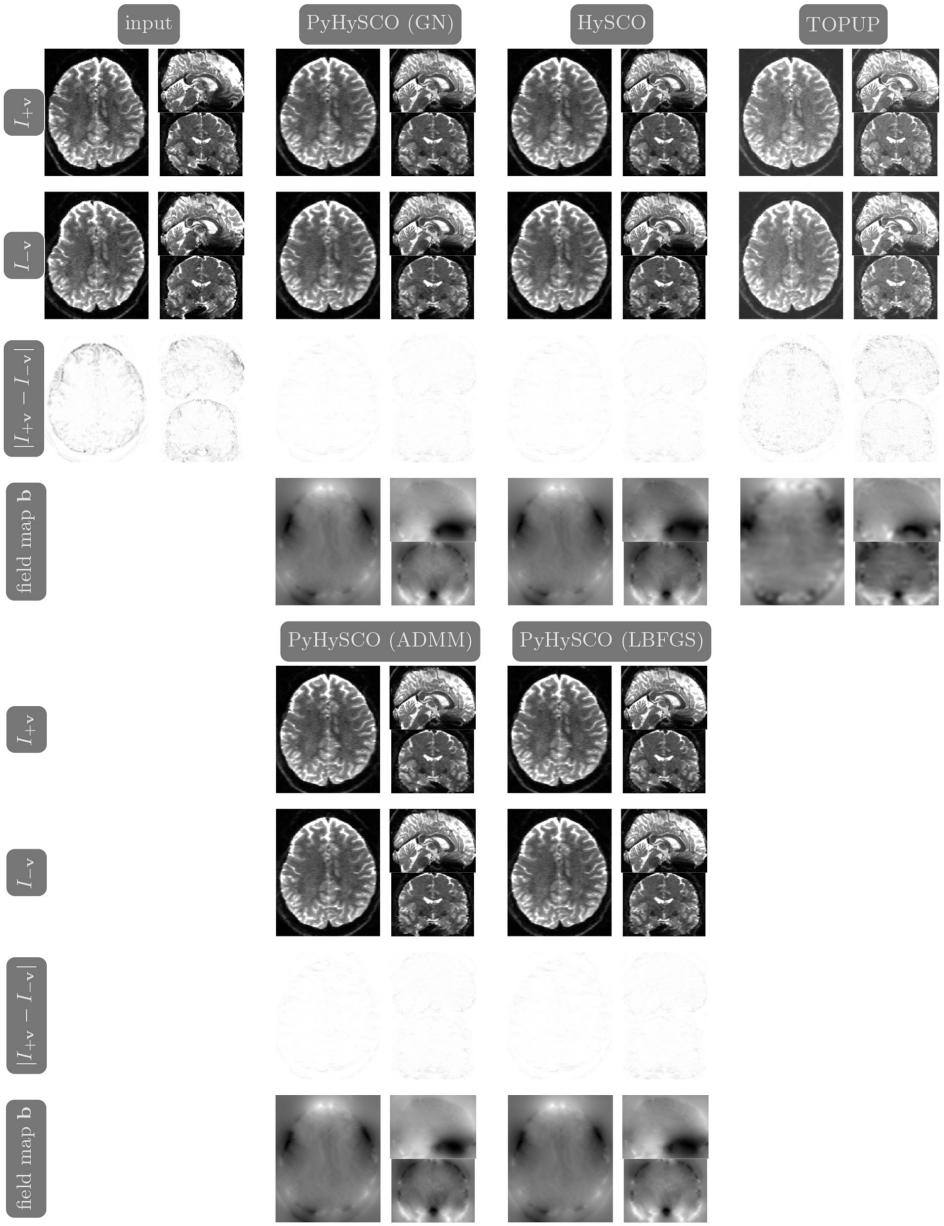
Visualization of resulting field maps and images for one subject from the 3T dataset (Subject ID: 211619). The first column in the top half shows the input data. The remaining columns show the results from PyHySCO using LBFGS, GN-PCG, and ADMM compared with TOPUP and HySCO. For each optimization, the top two rows are the pair of images with opposite phase encoding directions, and the third row shows the absolute difference (with inverted color) between the pair of images. The bottom row shows the field maps estimated for each method. PyHySCO achieves similar image distance and field map smoothness improvements in less computational time.

**Figure 8:**
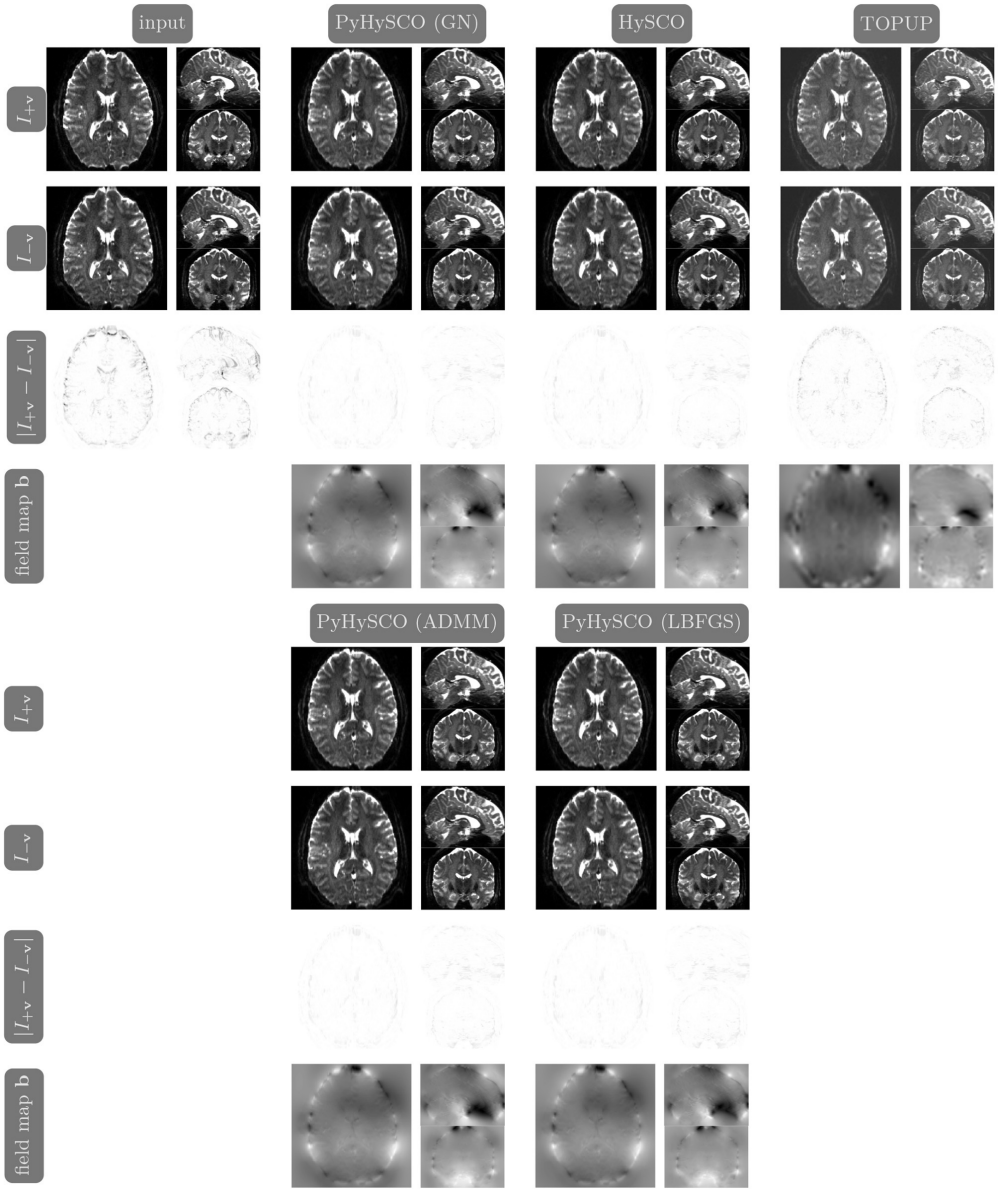
Visualization of resulting field maps and images for one subject from the 7T dataset (Subject ID: 825048). The first column in the top half shows the input data. The remaining columns show the results from PyHySCO using LBFGS, GN-PCG, and ADMM compared with TOPUP and HySCO. For each optimization, the top two rows are the pair of images with opposite phase encoding directions, and the third row shows the absolute difference (with inverted color) between the pair of images. The bottom row shows the field maps estimated for each method. PyHySCO achieves similar image distance and field map smoothness improvements in less computational time.

**Figure 9:**
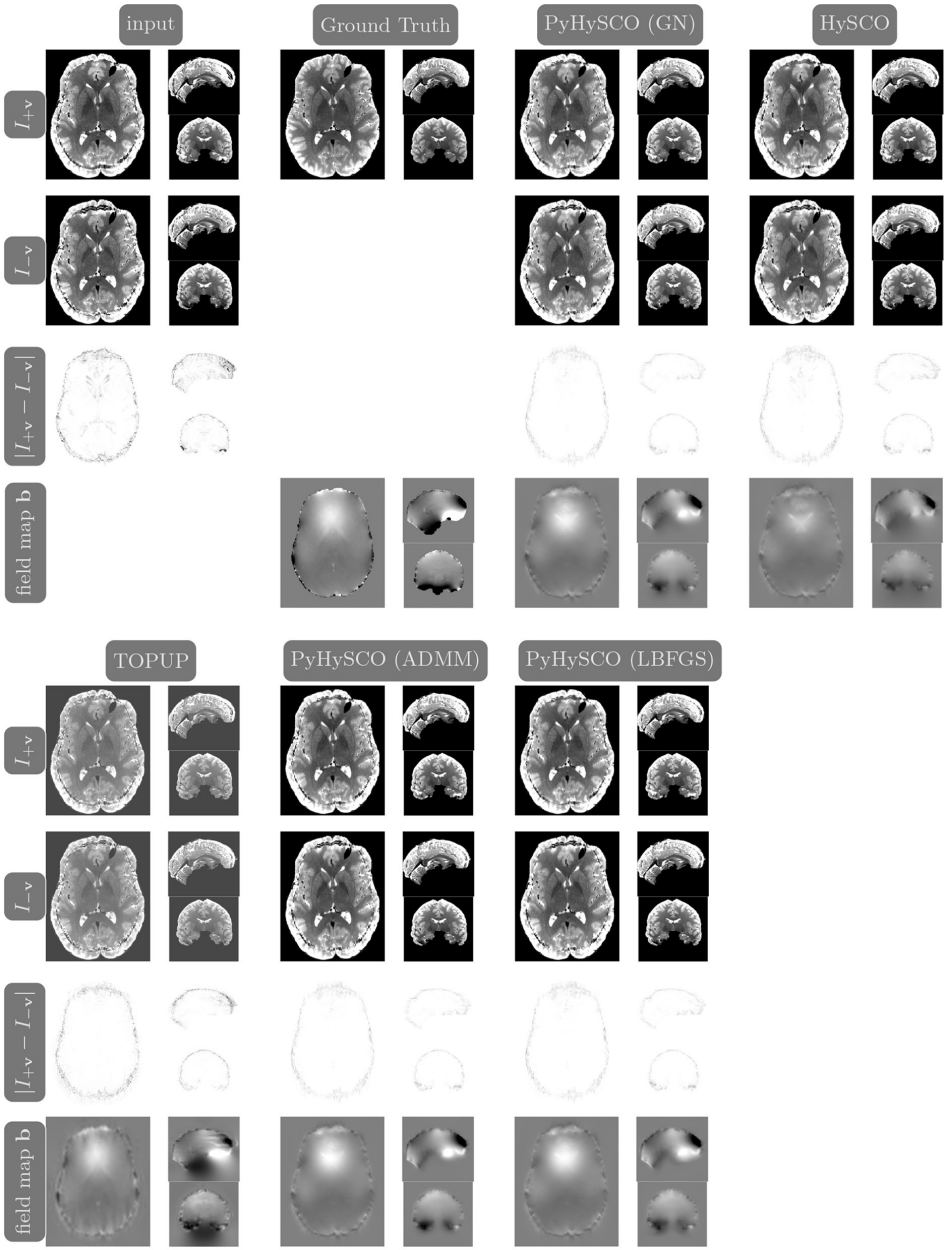
Visualization of resulting field maps and images for one subject from the simulated dataset (Subject ID: 105014). The first column in the top half shows the input data and the second column shows the ground truth T2w image and field map. The remaining columns show the results from PyHySCO using LBFGS, GN-PCG, and ADMM, TOPUP, and HySCO. For each optimization, the top two rows are the pair of images with opposite phase encoding directions, and the third row shows the absolute difference (with inverted color) between the pair of images. The bottom row shows the field maps estimated for each method. PyHySCO achieves similar image distance and field map smoothness improvements in less computational time.

We compare the results of PyHySCO using GN-PCG, ADMM, and LBFGS on both GPU and CPU architectures. Table 3 shows the runtimes and correction quality of each optimizer on CPU and GPU. All optimizers achieve a similar correction quality regarding a relative improvement of image distance, loss value, and smoothness regularizer value. However, GN-PCG has faster runtime on both CPU and GPU. On real data, GN-PCG took 10–13 s on average on GPU and 27–31 s on average on CPU, while ADMM took 11–15 s on GPU and 98–158 s on CPU, and LBFGS took 23–36 s on GPU and 104–141 s on CPU. Table 4 shows optimization metrics, including the number of iterations, stopping criteria, number of function evaluations, number of Hessian evaluations, and number of inner iterations if applicable. Consistent with its faster runtime, optimization with GN-PCG achieves a similar loss value with less computation as measured by objective function and Hessian evaluations. Figures 7–9 show the field map and corrected images of each optimizer for one example subject from each dataset. The field maps and corrected images are visually similar across optimizers.

**Table 3:** The speed and quality of optimization in PyHySCO on GPU and CPU with LBFGS, Gauss-Newton, and ADMM.

		**LBFGS**		**GN-PCG**		**ADMM**	
		**CPU**	**GPU**	**CPU**	**GPU**	**CPU**	**GPU**
3T	Runtime (s)	104.45	23.13	27.37	10.37	98.54	11.58
		±70.74	±4.61	±4.53	±0.87	±30.15	±2.23
	Opt. Time (s)	100.28	16.70	23.13	4.38	94.53	5.63
		±70.82	±4.49	±4.53	±0.68	±30.20	±2.15
	Relative	81.47	82.32	82.74	82.74	82.76	82.77
	Improvement	±3.71	±3.40	±3.50	±3.50	±3.31	±3.30
	Loss Value	7.90*e*07	2.56*e*07	2.56*e*07	2.56*e*07	3.09*e*07	3.10*e*07
		±7.99*e*07	±7.72*e*06	±7.69*e*06	±7.69*e*06	±8.51*e*96	±8.56*e*06
	Smoothness	2.13*e*05	3.72*e*04	3.85*e*04	3.85*e*04	5.62*e*04	5.65*e*04
	Reg. Value	±2.56*e*05	±1.18*e*04	±1.21*e*04	±1.21*e*04	±1.65*e*04	±1.71*e*04
7T	Runtime (s)	141.44	36.23	31.71	13.62	158.64	15.25
		±117.38	±7.76	±3.18	±2.38	±46.99	±3.15
	Opt. Time (s)	135.72	29.23	26.84	6.57	152.69	8.34
		±116.29	±7.88	±3.15	±2.30	±46.64	±2.91
	Relative	80.75	85.74	85.76	85.76	85.87	85.85
	Improvement	±6.91	±4.99	±5.10	±5.10	±4.99	±4.99
	Loss Value	2.25*e*08	4.25*e*07	4.14*e*07	4.14*e*07	4.43*e*07	4.43*e*07
		±2.22*e*08	±2.00*e*07	±1.95*e*07	±1.95*e*07	±1.99*e*07	±1.95*e*07
	Smoothness	6.38*e*05	6.00*e*04	5.63*e*04	5.63*e*04	6.68*e*04	6.66*e*04
	Reg. Value	±7.01*e*05	±2.18*e*04	±1.91*e*04	±1.91*e*04	±2.18*e*04	±3.68*e*04
Sim.	Runtime (s)	6344.93	143.77	1094.96	55.26	7687.28	52.72
		±649.21	±6.47	±135.20	±3.86	±4596.31	±18.01
	Opt. Time (s)	6320.43	125.95	1070.65	37.60	7662.55	35.15
		±649.01	±6.40	±135.69	±4.54	±4596.38	±17.92
	Relative	75.45	75.44	76.28	76.28	74.93	75.00
	Improvement	±5.40	±5.35	±5.19	±5.18	±5.59	±5.34
	Loss Value	6.03*e*07	6.00*e*07	6.08*e*07	6.08*e*07	6.08*e*07	6.12*e*07
		±1.44*e*07	±1.41*e*07	±1.40*e*07	±1.40*e*07	±1.40*e*07	±1.43*e*07
	Smoothness	9.06*e*04	8.94*e*04	9.56*e*04	9.56*e*04	8.97*e*04	9.12*e*04
	Reg. Value	±2.94*e*04	±2.74*e*04	±2.74*e*04	±2.72*e*04	±2.79*e*04	±2.77*e*04

We report for each dataset and optimizer the mean and standard deviation of total runtime (including loading and saving data), optimization time, improvement in distance between corrected images relative to input image, loss value, and smoothness regularizer value. Gauss-Newton achieves a similar correction quality in less time than LBFGS or ADMM on both CPU and GPU.

**Table 4:** Details of optimization for PyHySCO optimizers LBFGS, Gauss Newton, and ADMM.

		**LBFGS**	**GN-PCG**	**ADMM**
3T	Iterations	455.30	8.400	36.05
		±52.80	±0.92	±10.37
	Stopping Criteria (grad/loss/field map/max iter)	9/3/0/8	0/20/0/0	0/0/20/0
	Func. Evals	463.30	9.40	37.05
		±54.12	±0.92	±10.37
	Hessian Evals	N/A	92.40	437.50
			±10.08	±140.30
	Inner Iterations	N/A	10.0000	11.0269
			±0.00	±1.02
	Loss Value	2.56*e*07	2.56*e*07	3.10*e*07
		±7.72*e*06	±7.69*e*06	±8.56*e*06
7T	Iterations	405.00	7.50	56.75
		±65.61	±0.87	±17.01
	Stopping Criteria (grad/loss/field map)	14/3/0/3	0/20/0/0	0/0/20/0
	Func. Evals	415.35	8.50	57.75
		±68.00	±0.87	±17.01
	Hessian Evals	N/A	82.25	339.05
			±9.15	±101.55
	Inner Iterations	N/A	9.9722	4.9771
			±0.12	±0.08
	Loss Value	4.25*e*07	4.14*e*07	4.43*e*07
		±2.00*e*07	±1.95*e*07	±1.95*e*07
Simulated	Iterations	497.65	20.05	109.35
		±5.88	±1.83	±64.52
	Stopping Criteria	1/0/0/19	0/18/2/0	0/0/20/0
	(grad/loss/field map)
	Func. Evals	532.35	21.05	110.35
		±28.27	±1.83	±64.52
	Hessian Evals	N/A	220.55	1872.15
			±20.13	±1417.11
	Inner Iterations	N/A	10.0000	15.1681
			±0.00	±3.69
	Loss Value	6.00*e*07	6.08*e*07	6.12*e*07
		±1.41*e*07	±1.40*e*07	±1.43*e*07

For each dataset, we report the average and standard deviation number of iterations, count of stopping criteria used (gradient tolerance/ loss function change tolerance/ field map change tolerance/ maximum iterations), average and standard deviation number of function evaluations, average and standard deviation number of Hessian evaluations, average and standard deviation number of inner iterations, and average and standard deviation loss value. Gauss-Newton achieves a similar quality of correction with less computation than LBFGS or ADMM.

### 3.5 Single precision vs. double precision on GPU and CPU

We show the validity of PyHySCO using the Chang and Fitzpatrick initialization and GN-PCG in both double precision (64 bit) and single precision (32 bit) arithmetic on three different GPU architectures and a CPU architecture. These results are reported in Table 5. Since GPU architectures are optimized for the speed of lower precision calculations, we see a significant speedup when using single precision instead of double precision. However, there is a risk of lower accuracy or propagating errors when performing calculations in single precision, as it uses fewer bits to approximate floating point values. Empirically, we observed that the quality of our results is not significantly impacted by using single-precision arithmetic. We also observed consistent results across different GPU architectures: a Quadro RTX 8000, Titan RTX, and RTX A6000. Because PyHySCO is optimized to parallelize computations on GPU, the runtimes are faster on the GPUs compared to the Intel Xeon E5-4627 CPU.

**Table 5:** The speed and quality of PyHySCO optimization with Gauss-Newton on three different GPUs and a CPU in both single (float 32) and double (float 64) precision arithmetic.

		**RTX A6000**		**Quadro RTX 8000**		**Titan RTX**		**Intel Xeon E5-4627**	
		**(GPU)**		**(GPU)**		**(GPU)**		**(CPU)**	
		**Double**	**Single**	**Double**	**Single**	**Double**	**Single**	**Double**	**Single**
3T	Runtime (s)	12.7262	9.5750	13.4800	7.8854	13.1178	7.5820	34.3328	27.1305
		±0.68	±0.58	±1.23	±0.91	±1.31	±0.98	±4.26	±3.09
	Optimization	6.7947	4.1562	7.0133	2.1065	6.7862	1.9327	27.8682	23.2062
	Time (s)	±0.51	±0.39	±1.25	±0.90	±1.25	±0.62	±3.10	±3.07
	Relative	82.7486	82.7393	82.7486	82.7393	82.7486	82.7393	82.486	82.7393
	Improvement	±3.49	±3.50	±3.49	±3.50	±3.49	±3.50	±3.49	±3.50
	Loss Value	2.560*e*07	2.562*e*07	2.560*e*07	2.562*e*07	2.560*e*07	2.562*e*07	2.560*e*07	2.562*e*07
		±7.66*e*06	±7.69*e*06	±7.66*e*06	±7.69*e*06	±7.66*e*06	±7.69*e*06	±7.66*e*06	±7.69*e*06
	Smoothness	3.848*e*04	3.851*e*04	3.848*e*04	3.851*e*04	3.848*e*04	3.851*e*04	3.848*e*04	3.851*e*04
	Reg. Value	±1.21*e*04	±1.21*e*04	±1.21*e*04	±1.21*e*04	±1.21*e*04	±1.21*e*04	±1.21*e*04	±1.21*e*04
7T	Runtime (s)	17.2494	11.9028	21.0102	9.3140	18.6522	9.4680	82.1059	33.4020
		±0.94	±0.44	±6.31	±0.99	±2.84	±2.19	±7.64	±4.15
	Optimization	10.1298	5.7460	11.9937	2.2579	10.9617	2.9775	72.1380	28.5530
	Time (s)	±0.95	±0.42	±3.55	±0.64	±2.85	±2.11	±6.82	±4.09
	Relative	85.7618	85.7641	85.7618	85.7642	85.7618	85.7642	85.7618	85.7638
	Improvement	±5.10	±5.10	±5.10	±5.10	±5.10	±5.10	±5.10	±5.10
	Loss Value	4.143*e*07	4.140*e*07	4.143*e*07	4.140*e*07	4.143*e*07	4.140*e*07	4.1432*e*07	4.1410*e*07
		±1.95*e*07	±1.95*e*07	±1.95*e*07	±1.95*e*07	±1.95*e*07	±1.95*e*07	±1.95*e*07	±1.95*e*07
	Smoothness	5.634*e*04	5.628*e*04	5.634*e*04	5.628*e*04	5.634*e*04	5.628*e*04	5.634*e*04	5.629*e*04
	Reg. Value	±1.91*e*04	±1.91*e*04	±1.91*e*04	±1.91*e*04	±1.91*e*04	±1.91*e*04	±1.91*e*04	±1.91*e*04
Sim.	Runtime (s)	106.92	50.26	127.38	24.17	125.25	23.60	851.18	417.56
		±11.42	±3.52	±13.42	±1.31	±14.87	±3.78	±107.41	±55.33
	Optimization	89.53	35.53	104.47	7.93	105.78	9.32	827.06	402.04
	Time (s)	±11.56	±4.13	±13.88	±0.92	±14.84	±3.81	±108.91	±56.58
	Relative	76.27	76.28	76.27	76.28	76.27	76.28	76.27	76.28
	Improvement	±5.18	±5.18	±5.18	±5.18	±5.18	±5.18	±5.18	±5.18
	Loss Value	6.07*e*07	6.08*e*07	6.07*e*07	6.08*e*07	6.07*e*07	6.08*e*07	6.07*e*07	6.08*e*07
		±1.39*e*07	±1.40*e*07	±1.39*e*07	±1.40*e*07	±1.39*e*07	±1.40*e*07	±1.39*e*07	±1.40*e*07
	Smoothness	9.53*e*04	9.56*e*04	9.53*e*04	9.56*e*04	9.53*e*04	9.56*e*04	9.54*e*04	9.56*e*04
	Reg. Value	±2.71*e*04	±2.72*e*04	±2.71*e*04	±2.73*e*04	±2.71*e*04	±2.73*e*04	±2.71*e*04	±2.73*e*04

The relative improvement, loss value, and smoothness value are evaluated in double precision in all cases. The results are shown for both 3T and 7T data from the Human Connectome Project (Van Essen et al., 2012) and simulated data. There is a great speedup when calculating in single precision without losing the quality of correction, and the speedup of PyHySCO using a GPU is clear compared to the CPU.

### 3.6 A comparison of PyHySCO with HySCO and TOPUP

We compare the runtime, relative improvement, and resulting images after correction using PyHySCO to those given by TOPUP (Andersson et al., 2003) as implemented in FSL (Smith et al., 2004) using the default configuration,^2^ and HySCO (Ruthotto et al., 2013) as implemented in the ACID toolbox for SPM using the default parameters. HySCO is also based on the optimization problem Equation (6), while TOPUP uses a slightly different objective function. This makes it difficult to compute the smoothness and loss function values for TOPUP.

Table 6 reports the runtime and correction quality for PyHySCO using GN-PCG, HySCO, and TOPUP. On real 3T and 7T data, PyHySCO achieves lower loss and higher relative improvement between corrected images than HySCO and higher relative improvement than TOPUP. The runtime on CPU for real data is 1–2 min for HySCO and over 1 h for TOPUP, while PyHySCO on GPU has runtimes of 10–13 s. For the simulated dataset, PyHySCO requires an average of 1 min on GPU, HySCO requires an average of 12.6 min on CPU, and TOPUP requires an average of 8.5 h on CPU. Using the ground truth field maps from the simulated dataset, PyHySCO achieves the lowest average field map relative error of 14.48% compared to 19.70% for HySCO and 16.36% for TOPUP. PyHySCO also achieves the highest structural similarity (SSIM; Wang et al., 2004) with the ground truth field map of 91.80 compared to 86.91 for HySCO and 80.15 for TOPUP. All three methods average a structural similarity of over 99 with the ground truth T2-weighted image. Figures 7–9 show the field map and corrected images for one example subject from each dataset. The results of the methods are similar, and the resulting field maps are comparable to those of the existing tools, HySCO and TOPUP, while PyHySCO is considerably faster.

**Table 6:** The speed and quality of optimization for TOPUP, HySCO, and PyHySCO.

		**PyHySCO**	**HySCO**	**TOPUP**
3T	Runtime (s)	10.37	65.06	4022.56
		±0.87	±8.64	±73.11
	Relative	82.74	78.98	54.36
	Improvement	±3.50	±6.39	±17.08
	Loss Value	2.56*e*07	4.13*e*07	N/A
		±7.69*e*06	±1.38*e*07	
	Smoothness	3.85*e*04	7.84*e*04	N/A
	Reg. Value	±1.21*e*04	±3.01*e*04	
7T	Runtime (s)	13.62	120.92	3713.51
		±2.38	±19.61	±63.04
	Relative	85.76	80.43	74.51
	Improvement	±5.10	±10.46	±9.13
	Loss Value	4.14*e*07	5.87*e*07	N/A
		±1.95*e*07	±2.48*e*07	
	Smoothness	5.63*e*04	8.03*e*04	N/A
	Reg. Value	±1.91*e*04	±3.68*e*04	
Simulated	Runtime (s)	55.26	757.65	30854.18
		±3.86	±96.26	±568.11
	Relative	76.28	69.53	17.56
	Improvement	±5.18	±5.10	±28.14
	Loss	6.08*e*07	6.07*e*07	N/A
	Value	±1.40*e*07	±1.51*e*07	
	Smoothness	9.56*e*04	6.10*e*04	N/A
	Reg. Value	±2.72*e*04	±1.60*e*04	
	Relative Error	14.48	19.70	16.37
	(Field Map)	±7.71	±11.70	±3.60
	SSIM	91.80	86.91	80.15
	(Field Map)	±0.03	±0.05	±0.08
	SSIM	99.87	99.95	99.96
	(T2w Image)	±0.0017	±0.0003	±0.0002

PyHySCO uses Gauss Newton and optimizes in single precision on GPU. HySCO and TOPUP optimize on CPU using the default configurations. The results are reported for 3T and 7T data from the Human Connectome Project (Van Essen et al., 2012) and the simulated distortion data.

## 4 Discussion

The PyHySCO toolbox accurately and robustly corrects susceptibility artifacts in spin-echo EPIs acquired using RGP acquisition. In numerous experiments conducted with real and simulated data, PyHySCO achieves similar correction quality to leading RGP toolboxes, TOPUP and HySCO, while having a time-to-solution in the order of timings reported for pre-trained deep learning approaches. Compared to the latter class of methods, it is important to highlight that PyHySCO does not require any training and is based on a physical distortion model, which helps generalize to different scanners, image acquisition parameters, and anatomies.

PyHySCO's modular design encourages improvements and contributions. The toolbox is based on PyTorch, which provides hardware support and other functionality, including automatic differentiation. In our experiments, correction quality is hardware and precision-independent, but a considerable speedup is realized on GPUs with single precision (32-bit) arithmetic. The reduced computational time is mostly attributed to the effective use of multithreading and parallelism on modern hardware.

PyHySCO uses the one-dimensional correction of Chang and Fitzpatrick (1992) to initialize the non-linear optimization. In our numerical experiments, the scheme is fast and effective and we provide further insights through optimal transport theory. The initial estimate of the field map already substantially reduces the distance between the images with opposite phase encoding directions. In our experiments, the non-smoothness of the initial field map can be corrected by applying a Gaussian blur and a few optimization steps to the full image resolution.

The three optimization algorithms of PyHySCO achieve comparable correction results but have different computational costs. The ADMM algorithm takes advantage of the separable structure of the optimization problem to enhance parallelism but requires more iterations than GN-PCG. While this results in longer runtimes in our examples, the method could be more scalable for datasets of considerably higher resolution. For the relatively standard image sizes of about 200 × 200 × 132, the default GN-PCG algorithm is most effective. Both customized optimization algorithms are more efficient than our comparison, LBFGS.

PyHySCO can be interfaced directly in Python or run in batch mode via the command line. The latter makes it a drop-in replacement for other RGP tools in MRI post-processing pipelines.

The speed of PyHySCO relative to the existing tools makes it uniquely positioned to enable online distortion correction in applications where real-time decisions are necessary. For example, the speed of EPI acquisition along with the speed of PyHySCO distortion correction enables real-time distortion-free imaging useful for intra-operative guidance (see, e.g., Hall and Truwit, 2008; Roder et al., 2021; Yang et al., 2022). Additionally, PyHySCO can play a crucial role in the furthering of emerging fields such as fetal and neonatal imaging (see, e.g., Malamateniou et al., 2013; Afacan et al., 2019; Christiaens et al., 2019). In this application, EPI is popular for reducing the effects of uncontrollable subject motion, and fast distortion correction using PyHySCO can enable faster intervention if necessary.

## 5 Conclusion

PyHySCO offers RGP-based correction with high accuracy at a cost similar to pre-trained learning-based methods. Our implementation is based on PyTorch and makes efficient use of modern hardware accelerators such as GPUs. We show the accuracy and efficiency of PyHySCO on real and simulated three-dimensional volumes of various field strengths and phase encoding axes. Our results show that PyHySCO achieves a correction of comparable quality to leading physics-based methods in a fraction of the time.

## Data availability statement

Publicly available datasets were analyzed in this study. This data can be found at: https://www.humanconnectome.org. The source code, examples, and documentation for PyHySCO are available at the following repository: https://github.com/EmoryMLIP/PyHySCO. The Python package for PyHySCO can be installed via pip and be downloaded from: https://pypi.org/project/PyHySCO/.

## Ethics statement

Ethical approval was not required for the study involving humans in accordance with the local legislation and institutional requirements. Written informed consent to participate in this study was not required from the participants or the participants' legal guardians/next of kin in accordance with the national legislation and the institutional requirements.

## Author contributions

AJ: Investigation, Software, Validation, Visualization, Writing—original draft. LR: Investigation, Software, Writing—review & editing.

## Footnotes

^1^ https://github.com/hjmshi/PyTorch-LBFGS

^2^The default TOPUP configuration performs upsampling requiring the dimensions to be a multiple of 2. The configuration for TOPUP with images of the 3T data set does not perform upsampling due to the odd number of slices in the image volumes.

## References

[B1] Afacan O. Estroff J. A. Yang E. Barnewolt C. E. Connolly S. A. Parad R. B.. (2019). Fetal echoplanar imaging: promises and challenges. Top. Magnet. Reson. Imag. 28, 245–254. 10.1097/RMR.0000000000000219 31592991 PMC6788763

[B2] Alkilani A. Z. Çukur T. Saritas E. U. (2023). FD-Net: an unsupervised deep forward-distortion model for susceptibility artifact correction in EPI. arXiv preprint arXiv:2303.10436. 10.48550/arXiv.2303.10436 37811681

[B3] Andersson J. L. R. Skare S. Ashburner J. (2003). How to correct susceptibility distortions in spin-echo echo-planar images: application to diffusion tensor imaging. NeuroImage 20, 870–888. 10.1016/S1053-8119(03)00336-7 14568458

[B4] Antun V. Renna F. Poon C. Adcock B. Hansen A. C. (2020). On instabilities of deep learning in image reconstruction and the potential costs of AI. Proc. Nat. Acad. Sci. U. S. A. 117, 30088–30095. 10.1073/pnas.1907377117 32393633 PMC7720232

[B5] Bowtell R. McIntyre D. Commandre M. Glover P. Mansfield P. (1994). Correction of geometric distortion in echo planar images. Soc. Magn. Res. Abstr. 2:411.

[B6] Boyd S. Parikh N. Chu E. Peleato B. Eckstein J.. (2011). Distributed optimization and statistical learning via the alternating direction method of multipliers. Found. Trends Machine Learn. 3, 1–122. 10.1561/2200000016

[B7] Cai L. Y. Yang Q. Hansen C. B. Nath V. Ramadass K. Johnson G. W.. (2021). Prequal: an automated pipeline for integrated preprocessing and quality assurance of diffusion weighted MRI images. Magnet. Reson. Med. 86, 456–470. 10.1002/mrm.28678 33533094 PMC8387107

[B8] Chang H. Fitzpatrick J. M. (1992). A technique for accurate magnetic-resonance-imaging in the presence of field inhomogeneities. Med. Imag. IEEE Trans. 11, 319–329. 10.1109/42.158935 18222873

[B9] Chen Z. Pawar K. Ekanayake M. Pain C. Zhong S. Egan G. F. (2022). Deep learning for image enhancement and correction in magnetic resonance imaging–state-of-the-art and challenges. J. Digit. Imag. 9, 1–27. 10.1007/s10278-022-00721-9 36323914 PMC9984670

[B10] Christiaens D. Slator P. J. Cordero-Grande L. Price A. N. Deprez M. Alexander D. C.. (2019). In utero diffusion MRI: challenges, advances, and applications. *Top. Magnet. Reson. Imag*. 28, 255–264. 10.1097/RMR.0000000000000211 31592992

[B11] Clark I. A. Callaghan M. F. Weiskopf N. Maguire E. A. Mohammadi S. (2021). Reducing susceptibility distortion related image blurring in diffusion MRI EPI data. Front. Neurosci. 15:706473. 10.3389/fnins.2021.706473 34421526 PMC8376472

[B12] Cooley J. W. Lewis P. A. Welch P. D. (1969). The fast fourier transform and its applications. IEEE Trans. Educ. 12, 27–34.

[B13] Dávid G. Fricke B. Oeschger J. M. Ruthotto L. Fritz F. J. Ohana O.. (2024). Acid: a comprehensive toolbox for image processing and modeling of brain, spinal cord, and *ex vivo* diffusion MRI data. *BioRxiv*. 10.1101/2023.10.13.562027 PMC12290833 40800336

[B14] Duong S. Phung S. L. Bouzerdoum A. Taylor H. B. Puckett A. Schira M. M. (2020a). Susceptibility artifact correction for sub-millimeter fMRI using inverse phase encoding registration and T1 weighted regularization. J. Neurosci. Methods 336:108625. 10.1016/j.jneumeth.2020.108625 32061690

[B15] Duong S. T. Phung S. L. Bouzerdoum A. Schira M. M. (2020b). An unsupervised deep learning technique for susceptibility artifact correction in reversed phase-encoding EPI images. Magnet. Reson. Imag. 71, 1–10. 10.1016/j.mri.2020.04.004 32407764

[B16] Duong S. T. M. Phung S. L. Bouzerdoum A. Ang S. P. Schira M. M. (2021). Correcting susceptibility artifacts of MRI sensors in brain scanning: a 3D anatomy-guided deep learning approach. Sensors 21:72314. 10.3390/s21072314 33810289 PMC8037307

[B17] Esteban O. Daducci A. Caruyer E. O'Brien K. Ledesma-Carbayo M. J. Bach-Cuadra M.. (2014). “Simulation-based evaluation of susceptibility distortion correction methods in diffusion MRI for connectivity analysis,” in 2014 IEEE 11th International Symposium on Biomedical Imaging (ISBI) (Beijing: IEEE), 738–741.

[B18] Graham M. S. Drobnjak I. Jenkinson M. Zhang H. (2017). Quantitative assessment of the susceptibility artefact and its interaction with motion in diffusion MRI. PLoS ONE 12:e0185647. 10.1371/journal.pone.0185647 28968429 PMC5624609

[B19] Gu X. Eklund A. (2019). Evaluation of six phase encoding based susceptibility distortion correction methods for diffusion MRI. Front. Neuroinformat. 13:76. 10.3389/fninf.2019.00076 31866847 PMC6906182

[B20] Hall W. A. Truwit C. L. (2008). Intraoperative MR-guided neurosurgery. J. Magnet. Reson. Imag. 27, 368–375. 10.1002/jmri.21273 18183585

[B21] Hansen P. Nagy J. O'Leary D. (2006). Deblurring images: matrices, spectra, and filtering. Fundament. Algorit. 2006:74. 10.1137/1.978089871887418296179

[B22] Hestenes M. R. Stiefel E. (1952). Methods of conjugate gradients for solving linear systems. J. Res. Nat. Bur. Stand. 49, 409–436.

[B23] Holland D. Kuperman J. M. Dale A. M. (2010). Efficient correction of inhomogeneous static magnetic field-induced distortion in echo planar imaging. NeuroImage 50, 175–183. 10.1016/j.neuroimage.2009.11.044 19944768 PMC2819607

[B24] Hu Z. Wang Y. Zhang Z. Zhang J. Zhang H. Guo C.. (2020). Distortion correction of single-shot EPI enabled by deep-learning. NeuroImage 221, 117–170. 10.1016/j.neuroimage.2020.117170 32682096

[B25] Irfanoglu M. O. Modi P. Nayak A. Hutchinson E. B. Sarlls J. Pierpaoli C. (2015). Dr-buddi (diffeomorphic registration for blip-up blip-down diffusion imaging) method for correcting echo planar imaging distortions. Neuroimage 106, 284–299. 10.1016/j.neuroimage.2014.11.042 25433212 PMC4286283

[B26] Liu D. C. Nocedal J. (1989). On the limited memory BFGS method for large scale optimization. Math. Progr. 45, 503–528.

[B27] Macdonald J. Ruthotto L. (2017). Improved susceptibility artifact correction of echo planar MRI using the alternating direction method of multipliers. J. Math. Imag. Vis. 60, 268–282. 10.1007/s10851-017-0757-x

[B28] Malamateniou C. Malik S. Counsell S. Allsop J. McGuinness A. Hayat T.. (2013). Motion-compensation techniques in neonatal and fetal MR imaging. Am. J. Neuroradiol. 34, 1124–1136. 10.3174/ajnr.A3128 22576885 PMC7964586

[B29] Modersitzki J. (2009). FAIR: Flexible Algorithms for Image Registration, Vol. 6. Philadelphia, PA: Society for Industrial and Applied Mathematics.

[B30] Nocedal J. Wright S. J. (1999). Numerical Optimization. Berlin: Springer.

[B31] Paszke A. Gross S. Massa F. Lerer A. Bradbury J. Chanan G.. (2019). “Pytorch: an imperative style, high-performance deep learning library,” in Advances in Neural Information Processing Systems, eds. H. Wallach, H. Larochelle, A. Beygelzimer, F. d'Alché-Buc, E. Fox, and R. Garnett (Red Hook, NY: Curran Associates, Inc.), 32.

[B32] Penny W. D. Friston K. J. Ashburner J. T. Kiebel S. J. Nichols T. E. (2007). Statistical Parametric Mapping: The Analysis of Functional Brain Images. Cambridge, MA: Elsevier.

[B33] Peyré G. Cuturi M. (2017). Computational Optimal Transport. Center for Research in Economics and Statistics Working Papers, 2017-86. Hanover, MA: Now Publishers.

[B34] Roder C. Haas P. Tatagiba M. Ernemann U. Bender B. (2021). Technical limitations and pitfalls of diffusion-weighted imaging in intraoperative high-field MRI. Neurosurg. Rev. 44, 327–334. 10.1007/s10143-019-01206-0 31732818

[B35] Ruthotto L. Kugel H. Olesch J. Fischer B. Modersitzki J. Burger M.. (2012). Diffeomorphic susceptibility artifact correction of diffusion-weighted magnetic resonance images. Phys. Med. Biol. 57, 5715–5731. 10.1088/0031-9155/57/18/5715 22941943

[B36] Ruthotto L. Mohammadi S. Heck C. Modersitzki J. Weiskopf N. (2013). “Hyperelastic susceptibility artifact correction of DTI in SPM,” in Bildverarbeitung fuer die Medizin, eds. H.-P. Meinzer, T. M. Deserno, H. Handels, and T. Tolxdorff (Berlin; Heidelberg: Springer), 344–349 .

[B37] Saad Y. (2003). Iterative Methods for Sparse Linear Systems. Philadelphia, PA: SIAM.

[B38] Smith S. M. Jenkinson M. Woolrich M. W. Beckmann C. F. Behrens T. E. Johansen-Berg H.. (2004). Advances in functional and structural MR image analysis and implementation as FSL. Neuroimage 23, S208–S219. 10.1016/j.neuroimage.2004.07.051 15501092

[B39] Snoussi H. Cohen-Adad J. Commowick O. Combes B. Bannier E. Leguy S.. (2021). Evaluation of distortion correction methods in diffusion MRI of the spinal cord. arXiv [Preprint]. arXiv:2108.03817.

[B40] Stehling M. K. Turner R. Mansfield P. (1991). Echo-planar imaging: magnetic resonance imaging in a fraction of a second. Science 254, 43–50. 1925560 10.1126/science.1925560

[B41] Tax C. M. Bastiani M. Veraart J. Garyfallidis E. Irfanoglu M. O. (2022). What's new and what's next in diffusion mri preprocessing. NeuroImage 249:118830. 10.1016/j.neuroimage.2021.118830 34965454 PMC9379864

[B42] Van Essen D. C. Ugurbil K. Auerbach E. Barch D. Behrens T. E. Bucholz R.. (2012). The human connectome project: a data acquisition perspective. Neuroimage 62, 2222–2231. 10.1016/j.neuroimage.2012.02.018 22366334 PMC3606888

[B43] Wang Z. Bovik A. C. Sheikh H. R. Simoncelli E. P. (2004). Image quality assessment: from error visibility to structural similarity. IEEE Trans. Image Process. 13, 600–612. 10.1109/TIP.2003.819861 15376593

[B44] Wu M. Chang L.-C. Walker L. Lemaitre H. Barnett A. S. Marenco S.. (2008). “Comparison of EPI distortion correction methods in diffusion tensor MRI using a novel framework,” in International Conference on Medical Image Computing and Computer-Assisted Intervention (Berlin: Springer), 321–329. 10.1007/978-3-540-85990-1_39 PMC4819327 18982621

[B45] Yang J. Y.-M. Chen J. Alexander B. Schilling K. Kean M. Wray A.. (2022). Assessment of intraoperative diffusion EPI distortion and its impact on estimation of supratentorial white matter tract positions in pediatric epilepsy surgery. NeuroImage 35:103097. 10.1016/j.nicl.2022.103097 35759887 PMC9250069

[B46] Zahneisen B. Aksoy M. Maclaren J. Wuerslin C. Bammer R. (2017). Extended hybrid-space sense for EPI: off-resonance and eddy current corrected joint interleaved blip-up/down reconstruction. NeuroImage 153, 97–108. 10.1016/j.neuroimage.2017.03.052 28359788

[B47] Zahneisen B. Baeumler K. Zaharchuk G. Fleischmann D. Zeineh M. (2020). Deep flow-net for EPI distortion estimation. Neuroimage 217:116886. 10.1016/j.neuroimage.2020.116886 32389728

